# Human Herpesvirus 8 Interferon Regulatory Factor-Mediated BH3-Only Protein Inhibition via Bid BH3-B Mimicry

**DOI:** 10.1371/journal.ppat.1002748

**Published:** 2012-06-07

**Authors:** Young Bong Choi, Gordon Sandford, John Nicholas

**Affiliations:** Sidney Kimmel Comprehensive Cancer Center, Department of Oncology, Johns Hopkins University School of Medicine, Baltimore, Maryland, United States of America; University of Southern California Keck School of Medicine, United States of America

## Abstract

Viral replication efficiency is in large part governed by the ability of viruses to counteract pro-apoptotic signals induced by infection of host cells. For HHV-8, viral interferon regulatory factor-1 (vIRF-1) contributes to this process in part via inhibitory interactions with BH3-only protein (BOP) Bim, recently identified as an interaction partner of vIRF-1. Here we recognize that the Bim-binding domain (BBD) of vIRF-1 resembles a region (BH3-B) of Bid, another BOP, which interacts intramolecularly with the functional BH3 domain of Bid to inhibit it pro-apoptotic activity. Indeed, vIRF-1 was found to target Bid in addition to Bim and to interact, via its BBD region, with the BH3 domain of each. In functional assays, BBD could substitute for BH3-B in the context of Bid, to suppress Bid-induced apoptosis in a BH3-binding-dependent manner, and vIRF-1 was able to protect transfected cells from apoptosis induced by Bid. While vIRF-1 can mediate nuclear sequestration of Bim, this was not the case for Bid, and inhibition of Bid and Bim by vIRF-1 could occur independently of nuclear localization of the viral protein. Consistent with this finding, direct BBD-dependent inactivation by vIRF-1 of Bid-induced mitochondrial permeabilization was demonstrable *in vitro* and isolated BBD sequences were also active in this assay. In addition to Bim and Bid BH3 domains, BH3s of BOPs Bik, Bmf, Hrk, and Noxa also were found to bind BBD, while those of both pro- and anti-apoptotic multi-BH domain Bcl-2 proteins were not. Finally, the significance of Bid to virus replication was demonstrated via Bid-depletion in HHV-8 infected cells, which enhanced virus production. Together, our data demonstrate and characterize BH3 targeting and associated inhibition of BOP pro-apoptotic activity by vIRF-1 via Bid BH3-B mimicry, identifying a novel mechanism of viral evasion from host cell defenses.

## Author Summary

Viruses possess mechanisms of subverting host cell defenses against infection and virus replication; these mechanisms are essential to the virus life cycle. Here, we identify and characterize a novel mechanism of HHV-8 mediated inhibition of virus-induced programmed cell death (apoptosis). This function is specified by viral interferon regulator factor homologue vIRF-1, which binds to and directly inhibits pro-death activities of so-called BH3-only proteins (BOPs), induced and activated by stress signals such as those occurring in infected cells. The BH3 domains of BOPs mediate their pro-apoptotic functions, and it is these domains that are targeted by vIRF-1, via a region resembling a BH3-interacting and -inhibitory domain, termed BH3-B, present in one of the vIRF-1 targeted BOPs, Bid. The targeted BOP BH3 domains share characteristic and conserved features. As shown previously for Bim, depletion of Bid leads to enhanced HHV-8 productive replication, demonstrating that Bid, also, is a biologically significant negative regulator of virus replication and suggesting that its control by vIRF-1 is of functional importance. To our knowledge, this is the first report of viral targeting and inhibition of BOP activity via Bid BH3-B mimicry; our studies therefore expand the known mechanisms of viral evasion from antiviral defenses of the host.

## Introduction

Human herpesvirus 8 (HHV-8) specifies a number of proteins expressed during the lytic cycle that have demonstrated or potential abilities to promote virus productive replication via inhibition of apoptotic pathways induced by infection- or replication-induced stress. These proteins include membrane signaling receptors K1 and K15 [1]–[3], Bcl-2 and survivin homologues encoded by open reading frames 16 and K7 [4]–[7], viral chemokines vCCL-1 and vCCL-2 [8], and viral G protein-coupled receptor (vGPCR) [9], [10]. The viral interferon regulatory factor homologues, vIRFs 1–4, also are believed to play important roles in blocking interferon and other stress responses to virus infection and replication. Their functions include inhibitory interactions with cellular IRFs, IRF-activating pathways, and/or IRF-recruited p300/CBP transcriptional co-activators to IRF-stimulated promoters [11]–[15]. Additionally, the vIRFs inhibit apoptosis via targeting of other cellular proteins; these include p53 (vIRFs 1 and 3) [16]–[18], p53-activating ATM kinase (vIRF-1) [19], p53-destabilizing MDM2 (vIRF-4) [20], retinoic acid/interferon-inducible protein GRIM19 (vIRF-1) [21], and TGFβ receptor-activated transcription factors Smad3 and Smad 4 (vIRF-1) [22]. To date, the v-chemokines, vGPCR and vIRF-1 are the only HHV-8 proteins that have been demonstrated both to inhibit apoptosis in lytically infected cells and to promote HHV-8 productive replication, in the context of lytic reactivation in endothelial cells in the case of the vCCLs and vIRF-1 and additionally in primary effusion lymphoma (PEL) cells for vGPCR [23], [8], [10].

In addition to its other cellular binding partners, vIRF-1 also interacts with the pro-apoptotic BH3-only protein (BOP) Bim [23], a protein also targeted for suppression by v-chemokine signaling and demonstrated to be both induced during lytic replication and a very powerful negative regulator of viral replication efficiency [8]. Bim, like other BOPs, functions by virtue of its BH3 domain to target anti-apoptotic members of the Bcl-2 family and to disrupt their interactions with apoptotic executioner proteins Bax and Bak, liberating them for oligomerization and mitochondrial permeabilization [24], [25]. However, Bim can also interact with and activate Bax and Bak directly, via induced conformational changes [26]–[28]. This property of direct activation of Bax and/or Bak is shared by BOPs Bid and Puma, although other BOPs appear to act indirectly via BH3-mediated interactions with Bcl-2-family proteins [26], [27], [29], [30]. Activities of several BOPs, such as Bim, Bmf and Bad, are regulated via phosphorylation, to effect activation, inactivation, or alteration of protein stability [31]–[33]. For example, Bim is activated by JNK-mediated phosphorylation of residue T56, causing release of Bim from microtubules, inactivated by Akt phosphorylation of S87, which allows 14-3-3 association and cytosolic sequestration, and ERK phosphorylation of S69 to effect proteasomal degradation of Bim [34], [32], [35]. Bid is unique among BOPs in its activation via protease cleavage, typically by death receptor-activated caspases but also by other proteases, such as granzyme B [36]–[38]. Cleavage removes N-terminal sequences (p7) containing a motif, termed BH3-B, which interacts intramolecularly with the BH3 domain to inhibit Bid pro-apoptotic activity [39], [40]. Mitochondrial membrane targeting of cleaved, truncated Bid (tBid) is promoted via surface exposure of hydrophobic residues and N-terminal glycine myristoylation [41], [42]. While the nature of BH3 interactions with Bcl-2 family proteins, involving BH3 α-helix association with Bcl-2 BH1–3-comprised hydrophobic grooves, is well characterized [43], [44], the basis of Bid BH3∶BH3-B interaction is at present poorly understood [40].

Our previous studies of vIRF-1 interaction with Bim identified a unique mechanism of Bim regulation, via nuclear sequestration of the BOP away from mitochondria, and documented the first example of interaction between a Bcl-2 family member and an IRF homologue [23]. While the Bim-binding domain (BBD) of vIRF-1 was mapped, to residues 170–187 comprising a putative amphipathic α-helix, the region of Bim interacting with vIRF-1 was not determined. Subsequent comparisons of the BBD primary and predicted secondary structures with those of Bid BH3-B revealed similarities, in terms of conserved residues, α-helical structure and amphipathicity, indicating, by analogy, that BBD may target for binding and direct functional inhibition the BH3 domain of Bim (in addition to enabling vIRF-1-mediated inactivation of Bim via nuclear sequestration), and indeed may be able to bind Bid BH3 also. The data presented here demonstrate that this is indeed the case, that vIRF-1 can inhibit Bid as well as Bim pro-apoptotic activity, and that BBD can also recognize the BH3 domains of certain other BOPs, dependent on residues that are common and particular to these domains. Thus, vIRF-1 BBD mediates BH3-B mimicry, to our knowledge the first example of viral usurpation of this mode of inhibition of BOP function and pro-apoptotic signaling.

## Results

### Similarities between vIRF-1 BBD and Bid BH3-B

**Figure 1:**
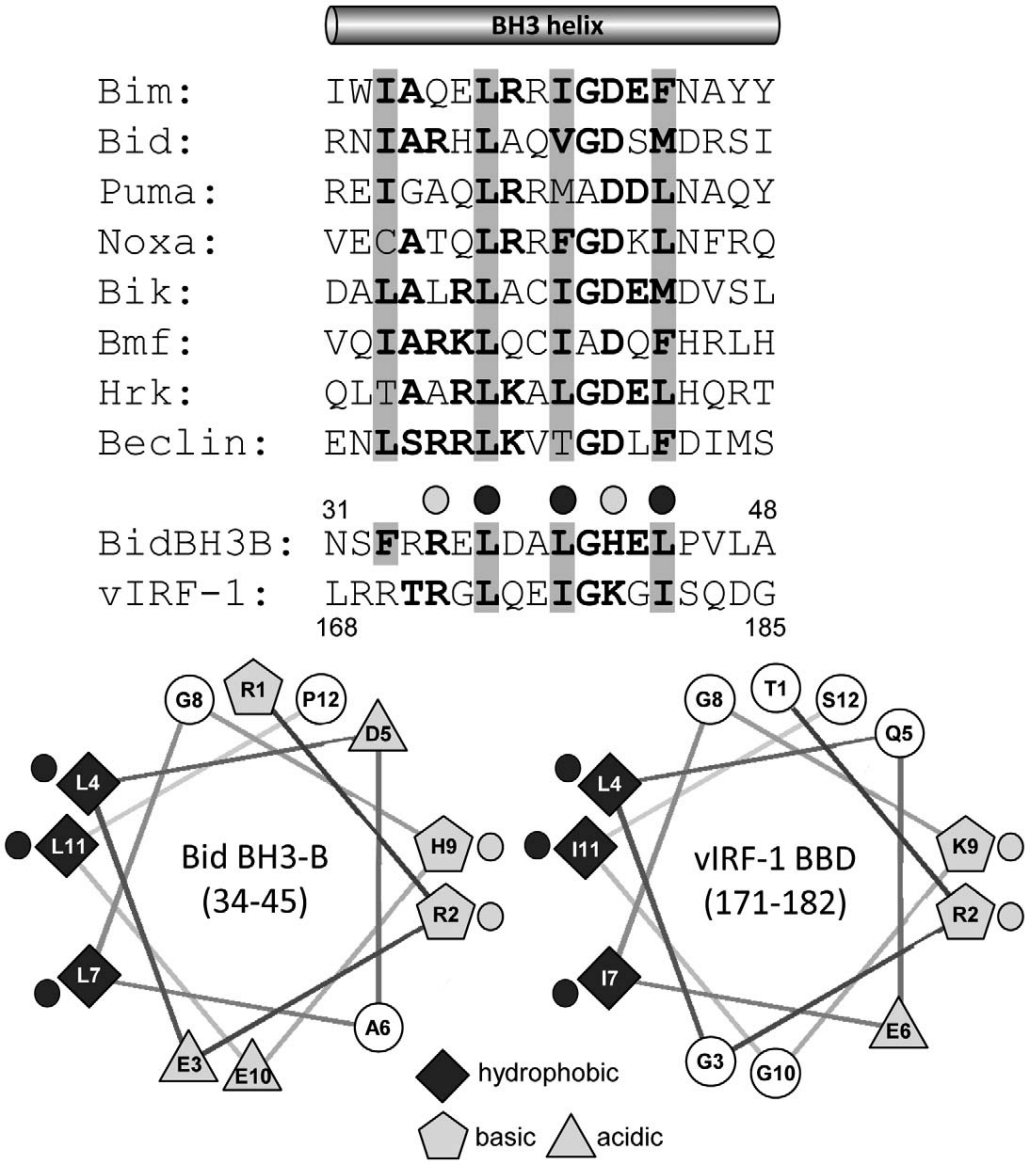
vIRF-1 BBD resembles the BH3-related BH3-B domain of Bid. Alignments of the primary sequence of vIRF-1 BBD with those of Bid BH3-B and BOP BH3 domains and comparison of the predicted secondary structure of BBD with the BH3-B α-helix. Collinear hydrophobic residues are indicated by grey shading. For BBD and BH3-B sequences and helical wheels, collinear hydrophobic and basic residues are indicated by darkly and lightly shaded circles, respectively. Matched shading on helical wheels indicates hydrophobic (diamonds) and hydrophilic [basic (pentagons) and acidic (triangles)] residues, respectively. Helical wheels were drawn using web-based software created by Don Armstrong and Raphael Zidovetzki.

We noted previously the amphipathic α-helical structure of the Bim-binding domain (BBD, residues 170–187) of vIRF-1 [23]. This type of structure also is apparent in the so-called BH3-B domain of Bid, which interacts intramolecularly with the BH3 domain to effect inhibition of Bid activity [40]. Indeed, the sequences of BBD and BH3-B are similar to each other and to BH3 domains of other proteins (Fig. 1). Particularly noteworthy are the BH3-conserved hydrophobic and BH3-B-conserved basic residues of the BBD core sequence (Fig. 1). The structural similarities of BBD and BH3-B suggested the possibility that BBD might interact with Bim via its BH3 domain and that vIRF-1 might target Bid (and possibly other BOPs) in addition to Bim.

### Bim and Bid BH3 targeting by vIRF-1

**Figure 2:**
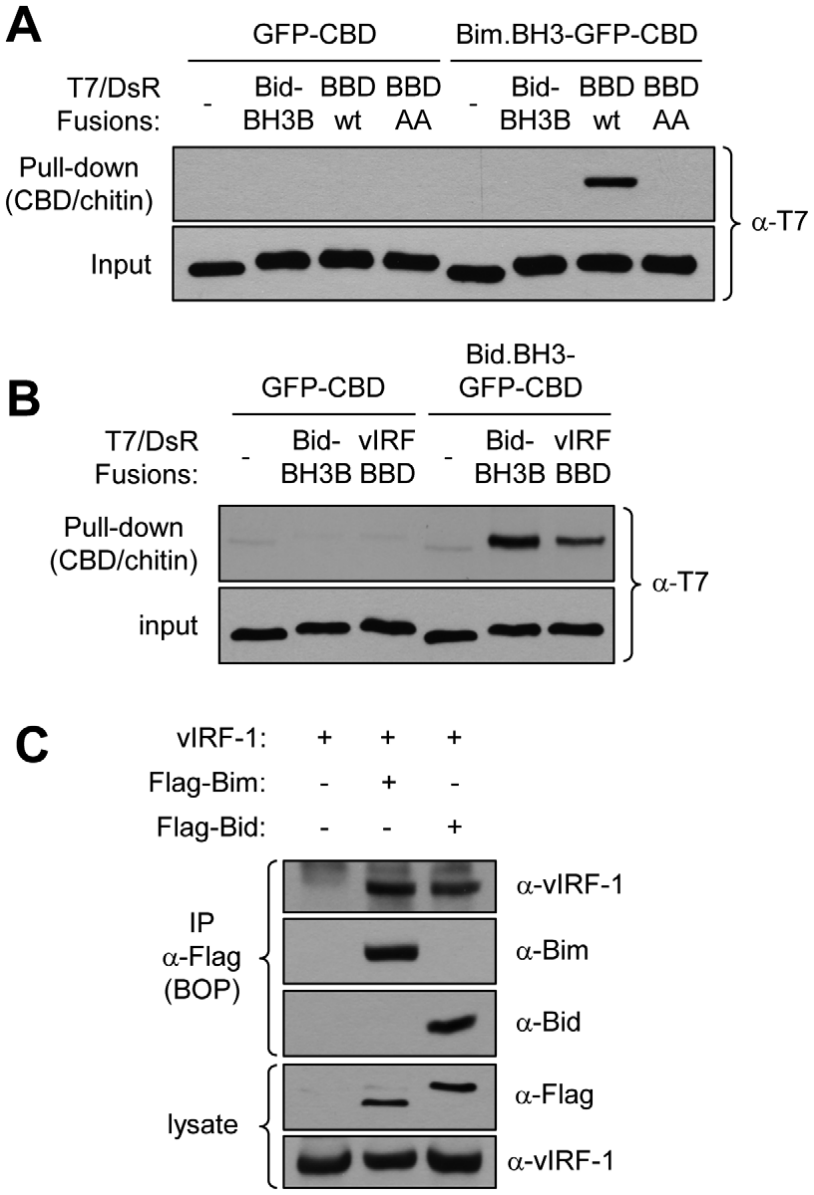
vIRF-1 BBD targets Bim and Bid BH3 domains. (A) *In vitro* co-precipitation assays were undertaken using bacterially-derived and purified recombinant vIRF-1 BBD and Bim BH3 domains fused to T7 epitope-tagged DsRed and GFP-chitin-binding domain (CBD), respectively. T7/DsRed-fused Bid BH3-B (Bid BH3-interacting) was also included, and BBD-AA (GK_179_AA, Bim BH3-refractory) and GFP-CBD were used as negative controls. Chitin bead-precipitated material was analyzed by immunoblotting to detect co-precipitated T7-tagged protein; wild-type BBD alone could be co-precipitated with Bim BH3-CBD. (B) Analogous experiments carried out using Bid BH3-GFP-CBD as “bait” identified similar interaction between BBD and Bid BH3, as detected also between Bid BH3-B and its cognate BH3 interaction partner (positive control). (C) Immunoprecipitation (IP) assays applied to transfected cell lysates were used to detect interactions between full-length vIRF-1 and Flag-tagged Bid_EL_ and Bim_EL_ [68], [69]. Co-precipitated vIRF-1 was detected by immoblotting using vIRF-1 antiserum, and α-Bim and α-Bid antibodies were used to confirm Flag antibody-mediated immunoprecipitation of the respective proteins. Immunoblotting for Flag and vIRF-1 verified appropriate expression of proteins in cell lysates.

To test whether the BBD of vIRF-1 interacted with the BH3 domain of Bim, recombinant fusion proteins were made for co-precipitation binding assays. The proteins comprised T7/DsRed-fused wild-type and Bim-binding-refractory GK_179_AA versions of BBD (vIRF-1 residues 170–187), and also Bid BH3-B (Bid_L_ residues 34–51), and chitin-binding domain (CBD)-tagged GFP-Bim BH3 (Bim_EL_ residues 148–161) and GFP (control). Paired T7/DsRed and GFP/CBD fusion proteins were mixed, CBD-tagged proteins precipitated with chitin beads, and precipitated material analyzed by SDS-PAGE and immunoblotting. This experiment revealed binding of BBD, but not BBD(GK_179_AA) or BH3-B, to Bim BH3, with no detectable background binding to negative control GFP-CBD (Fig. 2A). An analogous experiment using GFP/CBD-fused Bid BH3 (residues 86–99) as the “bait” identified interaction with Bid BH3-B (positive control) and also with vIRF-1 BBD (Fig. 2B), thereby identifying Bid BH3, as well as Bim BH3, as a target of vIRF-1 BBD interaction.

To confirm interaction of full-length vIRF-1 and Bid proteins, as we had done previously for vIRF-1 and Bim [23], appropriate expression plasmids were used to transfect HEK293T cells, and cell lysates were used for co-precipitation assays. Immunoprecipitation of Flag epitope-tagged Bid, as well as Bim (positive control), enabled co-precipitation of vIRF-1, demonstrating interaction between vIRF-1 and Bid (Fig. 2C).

### Functional equivalence of BBD and BH3-B

**Figure 3:**
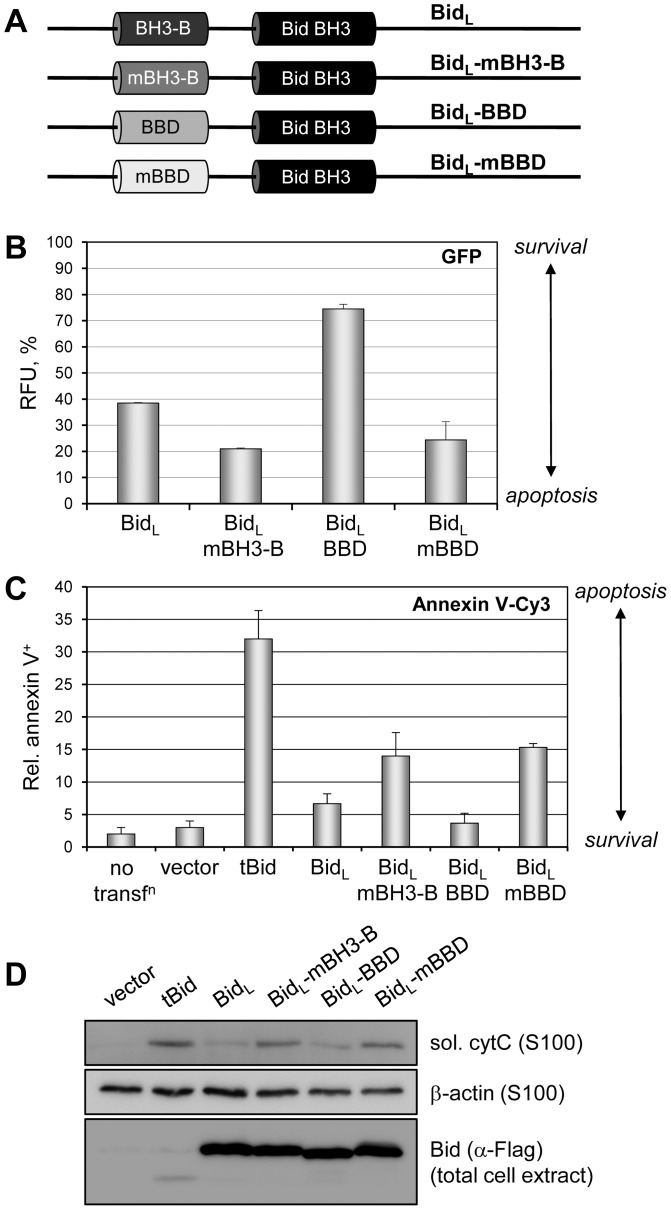
Functional equivalence of vIRF-1 BBD and Bid BH3-B. (A) Bid expression constructions were generated in which the BH3-B domain (Bid_L_ residues 33–48) was mutated [GHE_41_VLA (mBH3-B), BH3 refractory] or replaced with vIRF-1 BBD (residues 170–185) or substitution variant of BBD [GK_179_AA (mBBD)]. (B) These plasmids were individually cotransfected with GFP expression vector into HEK293T cells and after 24 h GFP fluorescence was quantified by fluorometry, providing a readout of cell viability. Relative fluorescence unit (RFU) values indicate the percentage of fluorescence relative to empty vector transfected cells (-Bid), with background, non-specific signal from untransfected cells (-GFP) subtracted. Error bars represent standard deviations from the average values derived from triplicate samples. (C) The functional equivalence of BH3-B and BBD in respect of apoptotic inhibition, specifically, was tested by using annexin V-Cy3 staining to detect HEK293T cells undergoing apoptosis at 7 h post-transfection. The inclusion of tBid expression plasmid and untransfected cultures in this experiment provided, respectively, a positive (BH3-B-deleted) control for induced apoptosis and a control for effects of transfection (by comparison to empty vector-transfected cultures). Cy3^+^ cells were counted from three random fields for each condition to generate the presented data; error bars show standard deviations from mean values obtained from individual fields. (D) Apoptotic inhibitory activity of vIRF-1 BBD in the context of Bid_L_ was further confirmed using a cytochrome c release assay. HEK293T cells were transfected with the indicated plasmids and harvested after 18 h. Dounce-derived extracts were either untreated or processed by centrifugation to derive total or S100 (soluble, sol.) samples for SDS-PAGE and immoblotting. Soluble sample blots were probed with antibodies specific for cytochrome c (cytC) or β-actin (loading control), and total cell extracts were probed with Flag antibody to detect and confirm expression of Flag-tagged Bid proteins.

The relationship between vIRF-1 BBD and Bid BH3-B was tested by substitution of the latter with the former in the context of full-length, uncleaved Bid (Bid_L_) and testing the constructions (Fig. 3A) for pro-apoptotic activities in appropriately transfected cells. Apoptotic activity of Bid was measured by a GFP-based assay, in which loss of GFP fluorescence in GFP vector-cotransfected cells correlates with loss of cell viability and corresponds to rates of apoptosis, e.g. as measured by TUNEL assay [23]. Transfection of Bid_L_ and GFP expression vectors into HEK293T cells led to substantially reduced GFP fluorescence (∼39%) relative to empty vector plus GFP control, set at 100% (Fig. 3B), consistent with previously reported apoptotic activity of uncleaved Bid [45], [40]. However, this activity was increased substantially by introduction of Bid-BH3 binding-abrogating mutations (GHE_41_VLA [40]) into BH3-B, suppressing GFP fluorescence to ∼20% [Fig. 3B, Bid(mBH3-B)]. Importantly, BBD could substitute fully for BH3-B in this assay, inhibiting BH3-mediated Bid apoptotic activity more effectively than native BH3-B (75% GFP fluorescence relative to 39%). Increased inhibition by BBD indicates that it may bind with higher affinity than BH3-B to Bid BH3. Although it is more likely that BBD and BH3-B mediated inhibition of Bid_L_ activity occur via intramolecular interactions with Bid BH3, it is also possible that trans-inhibition may occur. Introduction of mutation GK_179_AA (previously shown to abrogate Bim interaction [23]) into BBD (mBBD) abolished its inhibition of Bid_L_ activity, leading to GFP fluorescence (cell viability) levels similar to those obtained upon mutation of BH3-B.

Similar transfections with these constructions were undertaken to confirm apoptotic inhibitory activity of BBD in the context of Bid_L_. Annexin V-Cy3 staining (Fig. 3C) and cytochrome c release assays (Fig. 3D) were employed to quantify apoptosis and to monitor induction of the apoptotic pathway, respectively. The results derived from annexin V-Cy3 staining mirrored those obtained from the GFP-based assay (Fig. 3B), demonstrating full functional substitution of Bid BH3-B by vIRF-1 BBD. Apoptosis induced by tBid, included in this experiment, was notably higher than that of Bid_L_, Bid_L_-mBH3-B and Bid_L_-mBBD, as expected because of the complete absence of the inhibitory N-terminal region of Bid and efficient mitochondrial targeting of the truncated form. Congruent results were obtained from the cytochrome c release assays, confirming the ability of BBD to substitute functionally for BH3-B in the context of Bid_L_ (Fig. 3D).

Combined, the data presented in Figure 3 demonstrate functional equivalence of Bid BH3-B and vIRF-1 BBD and provide further evidence, in a biologically relevant context, of BBD interaction with Bid BH3.

### vIRF-1 effects on nuclear-cytoplasmic distribution of Bid

**Figure 4:**
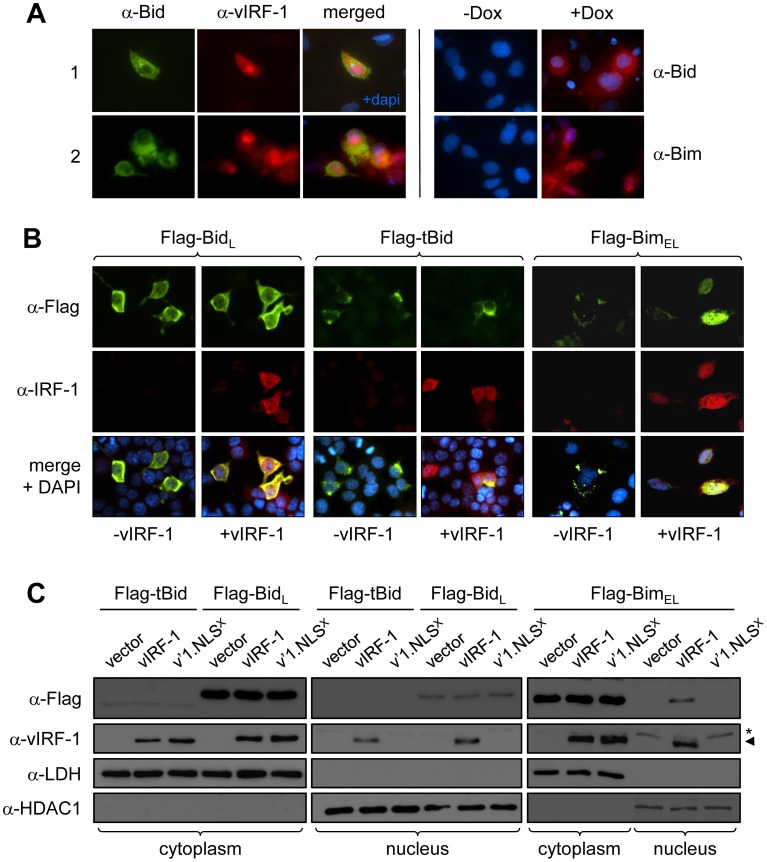
Bid localization in lytically reactivated and vIRF-1 transfected cells. (A) TIME-TRE/RTA cells were infected with HHV-8 and latency was allowed to establish. Cells were then reactivated by addition of 1 µg/ml doxycycline (Dox) to culture media and after 48 h cells were fixed and dually immunostained for detection of Bid and lytic antigen (vIRF-1). HHV-8^+^ TIME-TRE/RTA cells were also stained for detection of Bid or Bim in the absence and presence of Dox. Both BOPs were induced by Dox treatment (right panels), with general coincidence of Bid and lytic antigen immunofluorescence (left panels). Strong nuclear staining was evident only for Bim, with Bid localization remaining predominantly cytoplasmic. (B) HEK293T cells were transfected with expression vectors for Flag-tagged Bim_L_ or tBid and either empty vector (−vIRF-1) or vIRF-1 expression plasmid (+vIRF-1). Cells were immunofluorescence-stained to detect Flag (green) and vIRF-1 (red) and counterstained with DAPI to visualize nuclei (blue). Representative examples are shown. Nuclear localization of Bim but not Bid was induced by vIRF-1. (C) Nuclear and cytoplasmic extracts of similarly transfected cells were prepared and immunoblotted to provide independent analysis of potential vIRF-1 influence on Bid_L_ and tBid nuclear-cytoplasmic distribution. Extracts were prepared and fractionated as described in Materials and Methods and quality-checked by probing with cytoplasmic-localized lactate dehydrogenase (LDH) and nuclear-localized histone deacetylase 1 (HDAC1). Bim but not Bid relocalization in the presence of vIRF-1 co-expression was detected. Included in this experiment was a nuclear localization-defective variant of vIRF-1, vIRF-1.NLS^X^ (see Fig. 5 and associated legend and text), which was unable to induce Bim nuclear localization (v'1.NLS^X^ lane, α-Flag). (arrowhead, vIRF-1; asterisk, non-vIRF-1 α-Flag immunoreactive band).

In HHV-8 lytically reactivated endothelial cells, Bim is found predominantly in the nucleus, and nuclear location of Bim can be induced by vIRF-1 in transfected HEK293T cells [23]. As nuclear-localized Bim is inactive in respect of apoptotic induction, its nuclear sequestration represents a mechanism of BOP inactivation. To determine the nuclear-cytoplasmic distribution of Bid during HHV-8 lytic reactivation, dual-label immunofluorescence assays (IFA) were undertaken to identify Bid induction and distribution in reactivated cells expressing lytic antigen (vIRF-1). Like Bim, Bid was induced during lytic reactivation in telomerase-immortalized endothelial (TIME) cells [46], here engineered to express HHV-8 immediate-early protein RTA in response to doxycycline (see Materials and Methods and Fig.S1) (Fig. 4A). However, little or no nuclear localization of Bid was apparent, in sharp contrast to the predominant nuclear localization of Bim in lytically reactivated cultures [23] (Fig. 4A). In cells transfected with Bid_L_ or tBid expression vectors together with an empty or vIRF-1 expression plasmid, the nuclear-cytoplasmic distribution of each Bid protein was refractory to vIRF-1 influence (Fig. 4B). It is notable that some nuclear localization of Bid_L_ was apparent, consistent with previous reports of nuclear localization and associated activities of Bid [47]–[49], but no nuclear staining was evident for tBid. In contrast to Bid, and consistent with previous findings [23], Bim distribution was altered in the presence of vIRF-1, with strong nuclear staining apparent exclusively with vIRF-1 co-expression (Fig. 4B). That vIRF-1 indeed did not influence nuclear-cytoplasmic distribution of Bid was verified by using immunoblotting of cytoplasmic and nuclear fractions of transfected cells. Again, while nuclear localization of a proportion of Bid_L_ was detected, this was not detectably influenced by vIRF-1 co-expression, and tBid localization was restricted to the cytoplasm in the absence and presence of vIRF-1 (Fig. 4C). Furthermore, a nuclear localization-defective vIRF-1 variant (see below) also did not influence the nuclear-cytoplasmic distribution of Bid_L_, although induction of Bim nuclear localization was abolished (Fig. 4C).

### Nuclear translocation-independent inactivation of Bid and Bim

**Figure 5:**
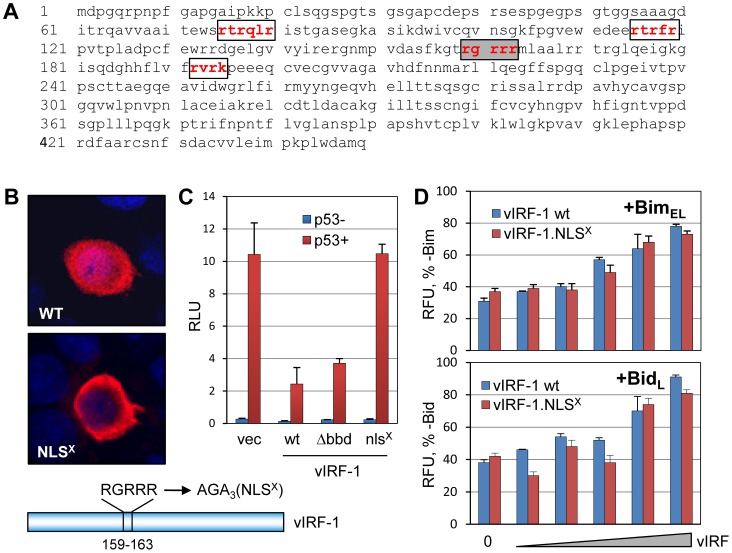
Nuclear localization-independent inhibition of Bim and Bid by vIRF-1. (A) Amino acid sequence of vIRF-1, showing putative nuclear localization signals (NLS, boxed), targeted for mutagenesis (basic-to-alanine residues). (B) Residues 159–163 (shaded box, panel A) were found to be required for nuclear localization of vIRF-1 in transfected HEK293T cells, as determined by immunofluorescence assay. Mutation of the other putative NLS sequences did not significantly affect vIRF-1 localization (data not shown). (C) Functional confirmation of nuclear exclusion of vIRF-1(RGRRR_163_AGAAA) (NLS^X^) using a p53-reporter assay, showing inhibition of nuclear-localized p53 activity by wild-type vIRF-1 (and also vIRF-1ΔBBD) but not by vIRF-1.NLS^X^. (D) Equivalent suppression of Bim and Bid apoptotic activity by wild-type and NLS-mutated vIRF-1, as determined by GFP-based cell viability assay applied to appropriately transfected HEK293T cells. For panels C and D, error bars represent standard deviations from the average values obtained from triplicate samples.

Targeting of BH3 domains of Bim and Bid by vIRF-1, coupled with the inability of vIRF-1 to induce significant Bid nuclear localization, suggested the possibility of direct inactivation of BOP apoptotic activity by vIRF-1 binding. To address this issue, we generated a nuclear localization-defective version of vIRF-1 for use in functional assays. Each of four potential nuclear localization signals (NLS) was mutated (Fig. 5A), and the respective vIRF-1 proteins were tested for nuclear localization in expression vector-transfected cells. vIRF-1(RGRRR_163_AGAAA) was found to be defective for nuclear localization, as determined by IFA (Fig. 5B). This was verified using a functional assay based on p53-inhibitory activity of vIRF-1; while wild-type vIRF-1 was able to suppress reporter-detected transactivation by nuclear p53, NLS-mutated vIRF-1 was completely inactive (Fig. 5C). As mentioned above, the vIRF-1 variant was also confirmed to be unable to induce nuclear localization of Bim (Fig. 4C). The wild-type and NLS-mutated versions of vIRF-1 were used in GFP-based viability assays to compare their abilities to inhibit Bim and Bid activities. Both vIRF-1 proteins were able to protect cells from Bim- and Bid-induced apoptosis, with very similar dose-activity profiles (Fig. 5D). Therefore, vIRF-1 inhibition of both Bim and Bid can be mediated independently of nuclear-localization and nuclear-localized functions of vIRF-1.

### Mitochondrial localization of vIRF-1

**Figure 6:**
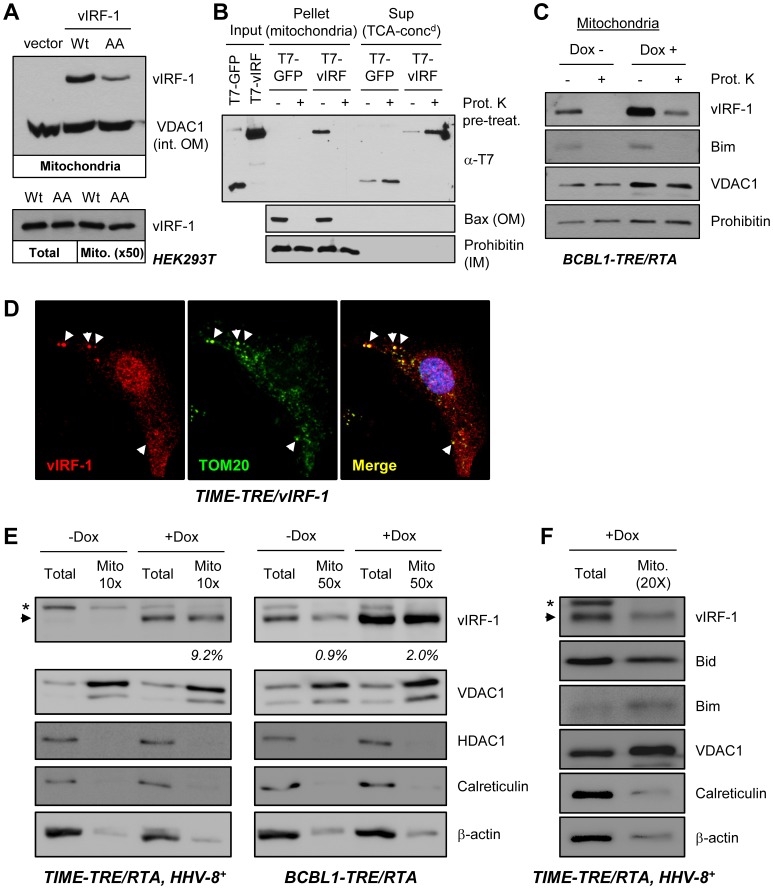
vIRF-1 localization to mitochondria. (A) Homogenates of HEK293T cells transfected with wild-type or BBD-mutated (GK_179_AA) vIRF-1 (or empty vector control, vIRF-1-negative) were subjected to differential and Optiprep gradient centrifugation to isolate enriched mitochondrial fractions (see Materials and Methods). These were analyzed by immunoblotting with vIRF-1-specific rabbit antiserum for the presence of vIRF-1 protein, and also with antibody to mitochondrial protein VDAC1 [voltage-dependent anion-selective channel protein 1; integral mitochondrial outer membrane (int. OM) protein] to provide a positive control. Normalization of vIRF-1 amounts in lysates versus mitochondrial fractions from these transfected cells was achieved using a ratio of 1∶50 (bottom). (B) *In vitro* mitochondrial binding assays using enriched mitochondria, untreated or pre-treated with proteinase K (Prot. K), and recombinant T7-tagged vIRF-1 for assessment of the requirement for surface protein integrity for mitochondrial binding by vIRF-1. Total protein released from mitochondria was precipitated with trichloroacetic acid (TCA) for direct quantitative comparison with protein from mitochondrial pellets. Blots were probed with antibodies to mitochondrial outer membrane (OM)-associated Bax and inner membrane (IM)-localized prohibitin to provide controls for appropriate fractionation, mitochondrial integrity, and proteinase K activity. (C) Generation and Western analysis of mitochondrial preparations from HHV-8^+^ BCBL-1-TRE/RTA cells [70] revealed mitochondrial association of endogenous vIRF-1, both in resting (latent) and lytically reactivated (+Dox) cultures; the latter, as expected, expressed higher levels of vIRF-1. In both cases, mitochondrial-associated vIRF-1 was susceptible to protease digestion, consistent with peripheral binding to mitochondria. Immunodetection of Bim verified peripheral protein susceptibility to proteinase K digestion. (D) Mitochondrial localization of vIRF-1 as determined using immunoflourescence assay for detection of vIRF-1 and mitochondrial marker TOM20 in TIME-TRE/vIRF-1 endothelial cells, +Dox. Arrows indicate examples of vIRF-1/TOM20 co-localization. (E) Western analyses of total cell and mitochondrial extracts of HHV-8^+^ TIME-TRE/RTA and BCBL1-TRE/RTA cells, untreated or treated with Dox for 2 days, were undertaken to quantify the relative amounts of mitochondrial-localized vIRF-1 in these latently and lytically infected cells. For TIME and BCBL-1 cells, 10- and 50-fold excess of mitochondrial extract over total extract, respectively, was loaded onto the gels to achieve near normalization. Relative signal intensities of bands were obtained from digitally captured images and calculated values of mitochondrial relative to total vIRF-1 levels are shown under the vIRF-1 blots. vIRF-1 was detected in latency (−Dox) only in the BCBL-1 (PEL) cells, and mitochondrial∶total vIRF-1 was increased upon Dox addition. Immunoblotting for VDAC1 (mitochondrial), histone deacetylase-1 (HDAC1, nuclear), calreticulin (endoplasmic reticulum) and β-actin (cytoplasmic) provided quality controls for mitochondrial and total cell extracts. (Arrowhead, vIRF-1; *non-specific). (F) A similar experiment in HHV-8^+^ TIME-TRE/RTA cells, demonstrating mitochondrial localization of Bid, in addition to vIRF-1, in lytically reactivated cultures.

To address the hypothesis that vIRF-1 may act directly at the mitochondrion to suppress BOP-induced apoptosis, we isolated mitochondria from vIRF-1 vector-transfected HEK293T cells and undertook SDS-PAGE and Western analysis for detection of vIRF-1 in the mitochondrial fraction. Both wild-type and Bim-refractory (GK_179_AA) vIRF-1 proteins were present in mitochondrial fractions, representing approximately 2% of total vIRF-1 present in the transfected cell lysates (Fig. 6A). To assess whether this vIRF-1 was likely to be membrane inserted/associated or mitochondrial outer membrane (OM) protein associated, *in vitro* mitochondrial binding assays were used. These assays employed purified recombinant vIRF-1 (T7 epitope-tagged) added to isolated mitochondria prior to and after proteinase K treatment. Binding of vIRF-1 to mitochondria, apparent absent treatment, was completely abrogated by proteinase K pre-treatment (Fig. 6B), indicating mitochondrial association of vIRF-1 via interaction with a cytoplasmic-exposed mitochondrial protein. Furthermore, endogenously-expressed vIRF-1 was present in mitochondrial fractions prepared from HHV-8^+^ primary effusion lymphoma (PEL) cells, BCBL1-TRE/RTA [50], with or without lytic induction (+Dox), and this vIRF-1 was susceptible to proteinase K digestion, demonstrating peripheral association of vIRF-1 with mitochondria in HHV-8 infected cells (Fig. 6C). vIRF-1 has been reported previously to be expressed during latency in PEL cells but to be induced during lytic reactivation [51], consistent with our detected patterns of vIRF-1 expression in BCBL1-TRE/RTA cells. Mitochondrial localization of vIRF-1 was verified by IFA in TIME-TRE/vIRF-1 cells [23] in which vIRF-1 expression is inducible upon addition of doxycycline to culture medium. These results demonstrated localization of detectable levels of vIRF-1 to a subset of loci staining positively for mitochondrial marker TOM20 (Fig. 6D). In both Dox-inducible BCBL1-TRE/RTA PEL cells and HHV-8-infected TIME-TRE/RTA endothelial cells (see Materials and Methods and Fig.S1), vIRF-1 was found by immunoblotting of mitochondrial fractions to localize in part to mitochondria during productive replication (Fig. 6E). The proportion of vIRF-1 localizing to mitochondria in the BCBL-1 cells was comparable with that measured in transfected cells (Fig. 6A); the level in the TIME cells (+Dox) was >4 times higher at 9%. For the PEL cells, which express vIRF-1 also during latency but at reduced levels, the proportion of mitochondrial-localized vIRF-1 was increased from 0.9% to 2% in Dox-treated, lytically-induced cultures, possibly reflecting biological significance of vIRF-1 activity at this site during productive replication. It should be noted that as only a subset of these cells support lytic reactivation, the proportion of vIRF-1 localized to mitochondria in lytically infected cells is likely to be substantially greater than the 2% level observed for the culture as a whole. Similar fractionation experiments in HHV-8^+^ TIME-TRE/RTA cells treated with Dox verified Bid, as well as vIRF-1, localization to mitochondria during lytic reactivation (Fig. 6F). Confocal immunofluorescence microscopy detected at least some colocalization of vIRF-1 and Bid in these cells (Fig.S2). Combined, our data provide evidence of mitochondrial association of vIRF-1, in both transduced and infected cells, and indicate that vIRF-1 may target BOPs for inhibition at this site.

### Direct inhibition of BOP activity by vIRF-1

**Figure 7:**
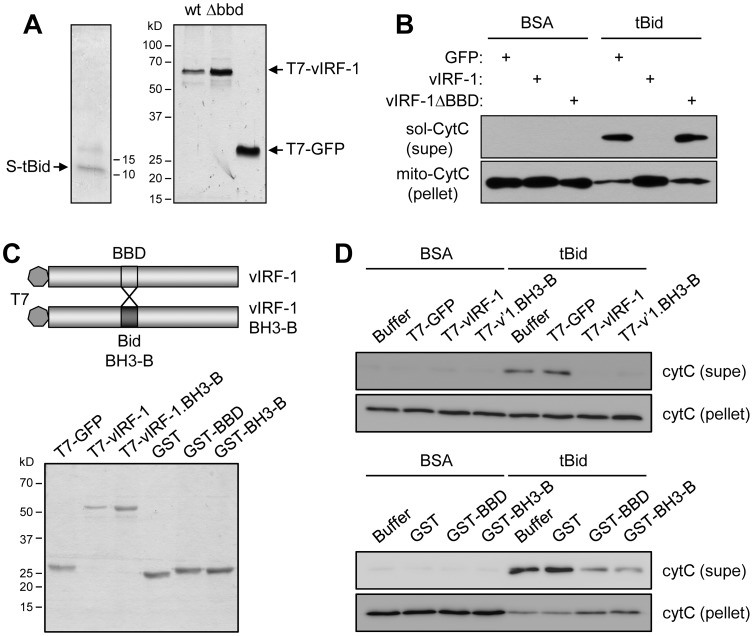
Direct functional inhibition of Bid by vIRF-1. (A) T7-fused vIRF-1, vIRF-1ΔBBD and GFP (negative control) and S-tag-fused tBid recombinant proteins (Materials and Methods) were checked for purity by SDS-PAGE and Coomassie staining. (B) Recombinant proteins were utilized in an *in vitro* mitochondrial permeabilization assay to determine the ability of vIRF-1 to suppress tBid-induced cytochrome c release from sucrose gradient-purified mitochondria (Materials and Methods). After 30 min. incubation, relative amounts of cytochrome c present in and released from mitochondria were determined by Western analysis of pellets and supernatants. Wild-type vIRF-1 (100 nM) completely blocked activity of tBid (applied at 100 nM) in this assay, whereas vIRF-1ΔBBD was inactive. (supe, supernatant; BSA, bovine serum albumin). (C) T7-vIRF-1 recombinant protein containing BH3-B in place of the BBD region was generated (shown diagrammatically) along with recombinant GST, GST-BBD and GST-BH3-B proteins. The purity and concentrations of these proteins were checked by SDS-PAGE and Coomassie staining. (D) As before, the proteins were utilized in *in vitro* mitochondrial permeabilization assays to determine their abilities to inhibit tBid-induced cytochrome c release. T7-vIRF-1.BH3-B and GST-fused BH3-B and BBD were all functional in this assay. Applied concentrations of tBid and vIRF-1 proteins were 100 nM (top); for tBid and GST-fusion proteins, the concentrations were 10 nM and 500 nM, respectively (bottom).

Bid and Bim BH3 domain-targeting by vIRF-1, nuclear localization-independent inhibition of BOP pro-apoptotic activity, and partial mitochondrial localization of vIRF-1 suggested the likelihood of direct BOP inactivation via BBD∶BH3 association. This was tested by using an *in vitro* mitochondrial permeabilization assay to assess the abilities of wild-type and a ΔBBD (BOP-refractory) variant of vIRF-1 to inhibit tBid-induced cytochrome c release. The vIRF-1 proteins and tBid were expressed as T7/intein/CBD and thioredoxin/His_6_/S-tag fusion proteins in bacteria and were subsequently purified and cleaved to release the respective T7- and S-tagged proteins (see Materials and Methods). SDS-PAGE and Coomassie staining (Fig. 7A) verified their purity prior to use. Addition of recombinant tBid (1.5 µg/ml, 100 nM) to mitochondrial preparations induced the release of cytochrome c into the soluble fraction of the mitochondrial suspension and led to a corresponding decrease in the level of cytochrome c in the mitochondrial pellet, as determined by Western analysis (Fig. 7B). Inclusion of vIRF-1 (8 µg/ml, 100 nM) blocked all detectable cytochrome c release, but vIRF-1ΔBBD was inactive in respect of tBid inhibition. These data demonstrate that BBD∶BH3 interaction alone is sufficient to inhibit tBid-induced apoptosis.

Further experiments were undertaken to determine if BH3-B could substitute functionally for BBD in the context of vIRF-1 to block tBid-induced mitochondrial permeabilization and also to confirm direct inhibitory activities of the BBD and BH3-B domains, expressed as fusions with GST. The recombinant proteins were isolated and purified from bacterial extracts and their purities and concentrations checked prior to experimental use (Fig. 7C). T7-fused wild-type vIRF-1 and its BBD-substituted counterpart each were able to inhibit tBid-induced cytochrome c release from mitochondrial preparations (Fig. 7D, top). Similarly, both GST-BBD and GST-BH3-B were able to inhibit tBid-induced cytochrome c release in this assay (Fig. 7D, bottom). These data confirm the functional equivalence of BBD and BH3-B, in the context of vIRF-1, and the direct role of these domains and their interaction with Bid BH3 in the inhibition of Bid pro-apoptotic activity.

### Range and specificity of BH3 recognition by vIRF-1 BBD

**Figure 8:**
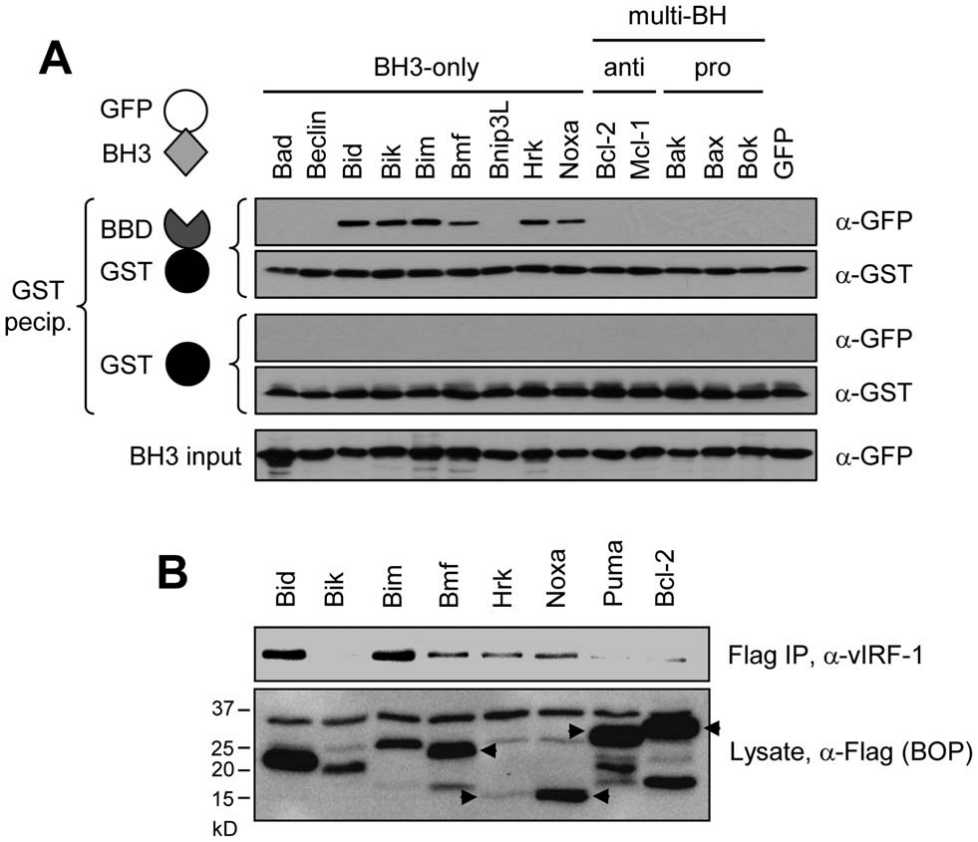
Additional targets of vIRF-1 BBD. (A) Recombinant proteins comprising GFP-fused BH3 domains of various BOPs and multi-BH-domain pro- and anti-apoptotic Bcl-2 family members were used together with GST-BBD (or GST, negative control) in co-precipitation assays, as illustrated. In addition to Bid and Bim, Bik, Bmf, Hrk and Noxa were co-precipitated with glutathione bead-sedimented GST-BBD (but not by GST alone). None of the non-BOP BH3 domains tested were co-precipitated. (B) Largely reflecting the *in vitro* binding data, Flag-tagged full-length BOPs containing the BBD-binding BH3 domains were, with the exception of Bik, able to co-precipitate vIRF-1 from lysates of HEK293T cells co-transfected with the appropriate expression vector pairs. Puma was included as an additional potential BOP target of vIRF-1. Bcl-2 served as a negative control. Arrows indicate bands corresponding to the full-length proteins of the expected sizes.

We next investigated whether BBD could target additional BH3 domains, in particular those of other BOPs. The respective BH3 coding sequences were cloned in-frame with the GFP open reading frame in plasmid vector pTYB4 and expressed in and purified from bacteria; BBD was expressed and isolated similarly, as a GST fusion protein (Materials and Methods). BH3 domains tested comprised those of BOPs Bad, Bmf, Bnip3L, Hrk and Noxa, along with Bim, Bid and “BH3-only” beclin [52], and BH3s from multi-BH domain proteins Bcl-2 and Mcl-1 (anti-apoptotic) and Bak, Bax and Bok (pro-apoptotic). Results from these co-precipitation assays identified Bik, Bmf, Hrk and Noxa BH3 domains as additional targets of BBD interaction (Fig. 8A). Interactions between the corresponding full-length proteins and vIRF-1 were tested by co-immunoprecipitation of vIRF-1 with Flag-tagged BOPs from transfected cell lysates; all but Bik were able to co-precipitate vIRF-1 in this experiment (Fig. 8B). Bcl-2 was essentially negative, with barely detectable levels of vIRF-1 in the co-precipitate, as was BOP Puma [not included in the BBD∶BH3 experiment (Fig. 8A)]. Therefore, of the Bcl-2 protein family members tested, BOPs Bim, Bid, Bmf, Hrk and Noxa are demonstrably targeted by vIRF-1, via BBD∶BH3 association, and Bik BH3 can also bind BBD.

### Determinants of BH3 recognition by vIRF-1 BBD

**Figure 9:**
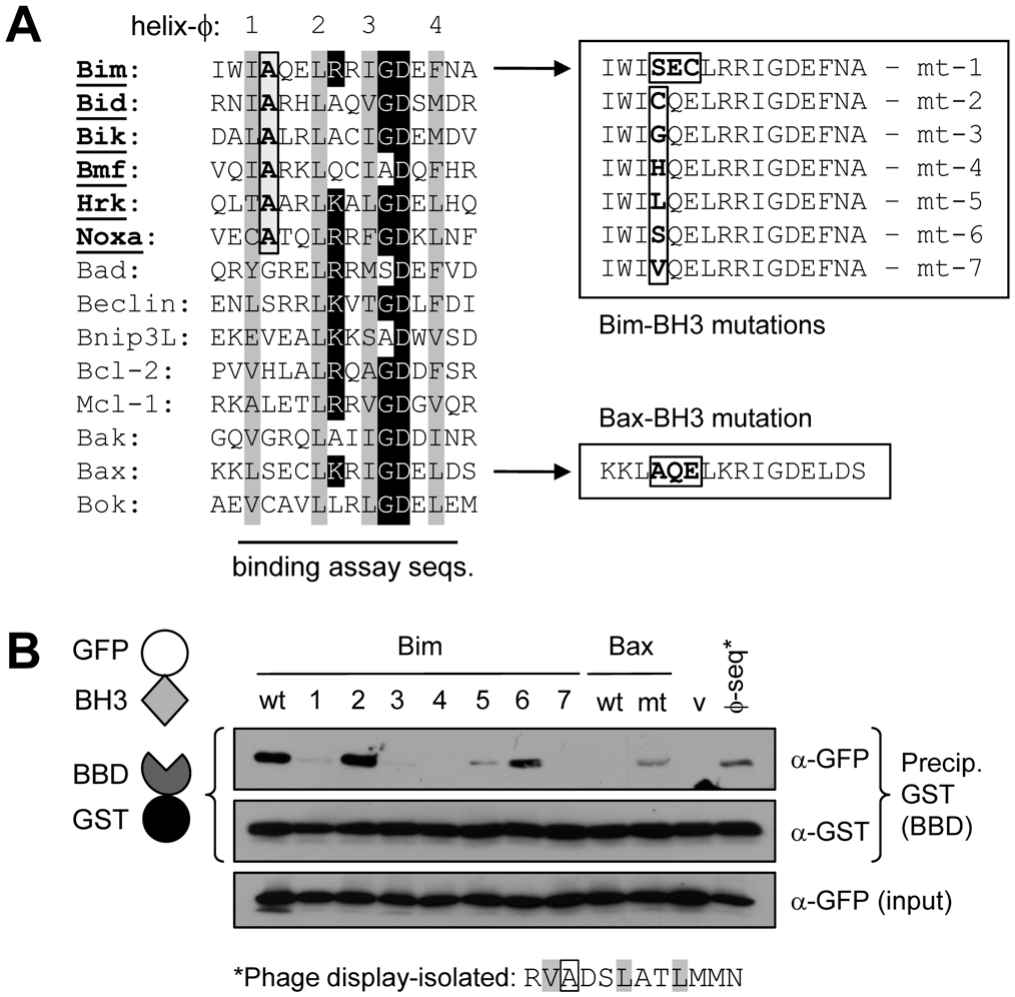
Structural analysis of BBD-BH3 interactions. (A) Substitutions were generated in Bim BH3 at position *φ*1+1 (boxed), containing a conserved alanine in all identified BBD-interacting BH3 domains, to match collinear residues in BH3 domains refractory to interaction with BBD. Mutual substitution of *φ*1+1 to *φ*1+3 residues in the BH3 domains of Bim and closely related Bax were also generated. The altered and native Bim and Bax BH3 sequences were cloned into a bacterial expression vector for generation of recombinant GFP-BH3 fusion proteins. Grey-shaded bars in the sequence alignment correspond to coaligned hydrophobic residues; black shading indicates conserved colinear amino acids. (B) *In vitro* co-precipitation assays utilizing recombinant GFP-BH3 and GST-BBD fusion proteins were utilized to analyze BH3∶BBD interactions. Lane labels correspond to wild-type (wt) and mutated Bim (1–7) and Bax (mt) BH3 sequences indicated in panel A; GFP alone (v, vector) was used as a negative control. A BH3-related peptide sequence isolated from a phage-display library using GST-BBD as bait (see Materials and Methods) was also included in the binding assay (*φ*-seq*).

Comparisons of the BH3 domains of vIRF-1/BBD-interacting BOPs identified a single unique and conserved residue among the BBD-binding BH3 sequences, namely an alanine at position *φ*1+1 (Fig. 9A, left). Mutagenesis of this position within the context of Bim BH3 was undertaken to determine its significance with respect to BBD binding; it was changed to each of the collinear residues of the non-binding BH3 domains. Additionally, residues *φ*1+1 to *φ*1+3 (SEC) of BBD-refractory Bax BH3 were changed to the equivalents (AQE) in closely related Bim BH3 and the reciprocal changes were made in Bim BH3 to determine if these “diverged” residues in combination could, respectively, confer and abrogate BBD binding. The various changes made are shown in Fig. 9A (right). As before, these sequences were expressed as GFP fusions for use in GST-BBD-based coprecipitation assays. Other than wild-type Bim BH3, glutathione bead-precipitated GST-BBD was able to efficiently co-precipitate only cysteine- and serine-substituted alanine *φ*1+1, with weak binding apparent for the leucine-substituted BH3 (Fig. 9B). The AQE substitution of Bax SEC residues was able to confer at least some BBD-binding capacity to Bax, demonstrating the contribution of these residues, either directly or via structural influence, to binding; the converse substitution in Bim abrogated binding. Interestingly, a sequence isolated from a phage-display dodecamer-peptide library using GST-BBD as bait had some resemblance to BH3 sequences in respect of conserved basic residues and, importantly, possessed an alanine residue at the equivalent of position *φ*1+1. This sequence also showed some binding in the *in vitro* coprecipitation assay. Taken together, these data indicate the likely central importance of alanine at position *φ*1+1 for BBD interaction, although the residue's contribution is likely indirect and evidently context dependent, as particular collinear small side-chain residues (serine and cysteine) from non-binding BH3 domains can substitute for alanine in Bim BH3 and the AQE motif from Bim can confer only weak binding to the closely related BH3 domain of Bax.

### Biological relevance of Bid to HHV-8 productive replication

**Figure 10:**
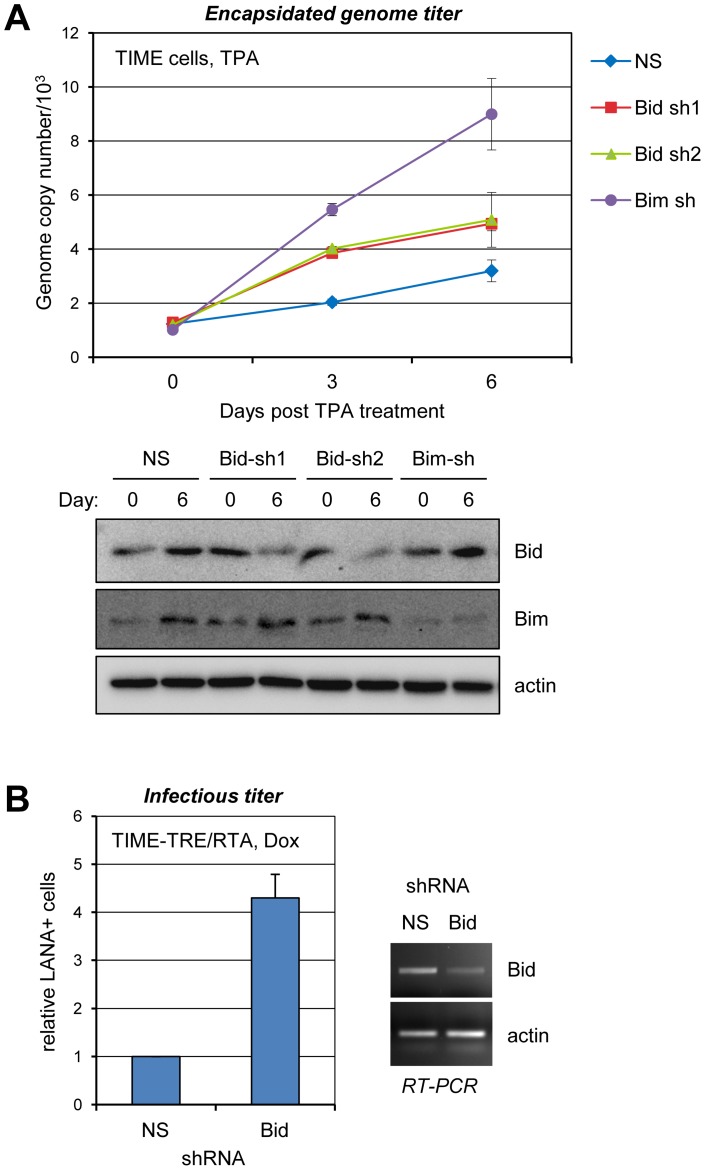
Biological significance of Bid in HHV-8 replication. (A) TIME cells latently infected with BCBL-1 PEL culture-derived virus were transduced with non-silencing (NS), Bid-specific (sh1, sh2), or Bim-specific shRNAs using lentiviral vectors (see Materials and Methods). After 48 h, these TIME cultures were treated with TPA to induce lytic reactivation, and media and cells were harvested at 0, 3 and 6 days post-induction for determinations of encapsidated genome copy numbers and Bid and Bim expression, respectively. Quantitative PCR was used to determine genome copies following pre-treatment with DNase I to remove any unencapsidated, contaminating viral DNA released from disrupted cells (see Materials and Methods). Immunoblotting confirmed both induction of Bid and Bim in induced cultures (detected even though a minority of cells support lytic replication [23], [8]) and their specific suppression by the respective transduced shRNAs. (B) Similar experiments were undertaken in control (NS shRNA-transduced) and Bid-depleted (Bid sh1-transduced) TIME-TRE/RTA cells using Dox (1 µg/ml) to induce lytic reactivation. Higher reactivation frequencies possible in these cells enabled relative virus titers from these cultures to be measured reliably by using an infectious assay, in which LANA^+^ cells were detected by immunofluorescence assay. Naïve TIME cultures were inoculated with ultracentrifuge-concentrated virus from Dox-treated HHV-8^+^ TIME-TRE/RTA cell culture media, cumulatively collected over 5 days following Dox addition. Numbers of infected, LANA^+^ TIME cells were counted from multiple random fields to derive average values. Results from two independent experiments are shown, with titers expressed relative to those obtained from NS shRNA-transduced cells (set at 1); the error bar represents deviation from the average NS/Bid titer ratios. Cells were harvested at day 5 for mRNA preparation and RT-PCR confirmation of Bid depletion (right).

Previous studies from this laboratory identified Bim as a potent inhibitor of virus productive replication and the importance of vIRF-1 BBD-mediated interactions in countering lytic cycle-induced apoptosis and promoting HHV-8 production in TIME cells [23], [8]. In view of the present findings that vIRF-1 inhibits Bid activity in a BBD-dependent fashion (Fig. 7) and that Bid is induced in lytically reactivated TIME cells (Fig. 4A), we wanted to test the significance of Bid in HHV-8 replication. To do this, we utilized lentiviral vector-delivered shRNAs directed to Bid mRNA sequences to deplete Bid in HHV-8^+^ (latently infected) TIME cells and compared levels of cell-released encapsidated viral genomes produced following TPA induction to those obtained from non-silencing (NS) shRNA-transduced control cultures. Bim shRNA-transduced TIME cells were also included to provide a positive control for the experiment. The data from this experiment (Fig. 10A) revealed that each of the two Bid shRNAs led to small but significant increases in virus production from TPA-treated TIME cells; as before, Bim depletion led to substantial amplification of virus titers. Similar experiments were undertaken in TIME-TRE/RTA cells to confirm the effect on replication of Bid depletion; here, lytic replication was induced by addition of doxycycline to the cultures. Again, Bid depletion led to increased virus production (Fig. 10B), measured in this experiment by titration of released infectious virus in culture media via inoculation of naïve TIME cells and detection of virus infection by immunofluorescence assay for latency-associated nuclear antigen (LANA). These data demonstrate that Bid, in addition to Bim, is a contributor to negative regulation of HHV-8 infection and suggest that its control by vIRF-1 is likely to be important for optimal virus productive replication. It is important to note, however, that the positive effects on virus replication of Bid and Bim depletion by shRNA transduction demonstrate also that vIRF-1 is not completely effective at suppressing the activities of these BOPs. This situation is not unexpected and is probably universal amongst such viral regulators of cellular activities.

## Discussion

Viruses have numerous and diverse mechanisms of apoptotic inhibition, necessitated by the pro-apoptotic signals induced upon *de novo* infection of a cell and by the processes associated with virus replication (reviewed in [53]–[55]). These mechanisms include inhibition by various means of interferon induction and signaling and inactivation and suppression of p53, pro-apoptotic proteins such as BOPs, and caspase mediators of apoptotic signaling. Examples are p53-inhibitory activities of simian virus 40 T-antigen and adenovirus E1B-55K [56], [57], caspase inhibition by HHV-8 specified K7/vIAP and gammaherpesvirus viral FLICE inhibitory (vFLIP) proteins [6], [7], [58], [59], and the Bcl-2 homologues specified by herpesviruses (gammaherpesvirus vBcl-2s and human cytomegalovirus UL37x1/vMIA), poxviruses (e.g., fowlpox virus 039), African swine fever virus (A179L), adenovirus (E1B-19K), and others [60], [61]. These viral Bcl-2 homologues, while not necessarily readily identifiable at the amino acid sequence level, have been demonstrated or are believed to preserve the essential BH-like helical domains and overall three-dimensional hydrophobic groove structure of cellular Bcl-2 proteins to allow their interactions with and inhibition of pro-apoptotic BH3-only proteins and/or apoptotic executioners Bax and Bak. Thus, Bcl-2 “mimicry” is a commonly used mechanism of viral evasion from innate host cell defenses via apoptotic induction. However, alternative mechanisms of direct BOP inhibition by viral proteins have not previously been reported, to our knowledge.

HHV-8 specifies a number of proteins that have predicted abilities to inhibit apoptosis induced by virus *de novo* infection and lytic replication and therefore have the potential to promote virus infection and establishment of latency and/or productive replication [62]. However, demonstration of such activities in the context of virus infection is largely lacking, although vIRF-1, in part via BBD-dependent interactions, has been found to promote cell survival under lytic-induced stress and to enhance HHV-8 productive replication in culture [23]. Our previous studies identified Bim as a potent negative regulator of HHV-8 productive replication, mapped the binding domain (BBD, residues 170–187) involved in its inhibition, and noted induced nuclear localization of Bim as a means of its inactivation by vIRF-1 to promote HHV-8 replication and cell survival [23], [8]. However, the region of Bim interacting with vIRF-1 was not mapped and the possibility of direct inhibition of Bim activity, in addition to inhibition via vIRF-1-induced nuclear sequestration, was not considered.

The present identification of BH3 as the target of BBD binding to Bim, as well as Bid and other BOPs, and finding of direct inactivation by vIRF-1 of Bid-induced mitochondrial permeabilization *in vitro*, Bim and Bid inhibition by nuclear localization-defective vIRF-1, and inability of vIRF-1 to induce significant Bid (Bid_L_, tBid) nuclear localization, demonstrate that vIRF-1 can inhibit BOPs independently of nuclear translocation. Indeed, in contrast to Bim, [23], nuclear localization of Bid was not apparent in lytically infected cells (Fig. 4). The mechanism of induced nuclear translocation of Bim could comprise cytoplasmic-to-nuclear chaperoning by vIRF-1 and/or nuclear capture of Bim translocating independently or by other means. The latter would be analogous to HHV-8 latency-associated nuclear antigen (LANA)-induced nuclear localization of predominantly cytoplasmic GSK3β [63], for example. The fact that Bid has been reported to localize in part to the nucleus, and indeed to function here as a component of the DNA repair machinery and as an apoptotic mediator in response to DNA damage [47]–[49], indicates that simple “nuclear capture” by vIRF-1 is unlikely to be a mechanism of vIRF-1-induced nuclear sequestration of Bim, as this would be expected to operate for Bid also. Specificity of Bim nuclear chaperoning by vIRF-1 is, similarly, difficult to explain, as Bim and Bid interactions with vIRF-1 occur by the same means (BH3∶BBD binding) and these BOPs, able to move between cellular compartments, appear to be equally susceptible to translocation. It is possible that while both BOPs can enter the nucleus, independently or promoted by vIRF-1, only Bim can, via its association with vIRF-1, form stable interactions with other nuclear proteins to effect its sequestration in this compartment. In trying to resolve this issue, it would be informative to determine whether vIRF-1 can induce nuclear translocation of any of the other BBD-targeted BOPs or if this activity is restricted to Bim. Regardless of mechanism, however, it is apparent that vIRF-1-induced nuclear sequestration of Bim represents a mechanism of inactivation of its pro-apoptotic activity, in addition to its direct inactivation via BH3 binding. It is possible that this is necessary for biologically sufficient inhibition of this powerful negative regulator of HHV-8 productive replication. It is also conceivable that there are as-yet unrecognized nuclear functions of Bim that contribute to HHV-8 lytic replication.

An important finding of the present study is that vIRF-1 can interact, via its Bid BH3-B-like BBD region, with the BH3 domains of BOPs Bid, Bik, Bmf, Hrk and Noxa in addition to Bim BH3, while refractory to interaction with other tested Bcl-2 family members (other BOPs and multi-BH-domain proteins). Thus, the “Bim-binding domain” of vIRF-1 should more appropriately be referred to as the “BOP-binding domain”. These additional interactions of BBD not only identify multiple new BOP interaction partners of vIRF-1 that could be targeted for inhibition, of likely relevance in the context of HHV-8 biology, but also provide the tools to better understand the molecular basis and specificity of BBD and BH3-B interactions with BH3 domains. Previous studies of the latter identified, via mutagenesis of murine Bid BH3-B sequences and analysis of BH3-B∶BH3 interaction and Bid activity, residues L_35_ (first hydrophobic, BH3/BBD-conserved) and G_39_, R_40_ and/or E_41_ (GHE in human Bid) as important for BH3-B∶BH3 binding *in vitro* and for both physical and functional interactions, respectively [40]. Our own *in silico*, mutagenesis, and binding studies indicate that for BBD∶BH3 interactions, an alanine residue corresponding to BH3 position “*φ*1+1” (Fig. 9A) is both conserved and specific to all BBD-interacting BOP BH3 domains and important for BBD∶BH3 interaction. An alanine at this position was also identified in BH3-like sequences [R*V* ADS*L*AT*L*MMN (Fig. 9B), S*I* ANT*I*AS*V*QFM, and I*F* AALDYN*L*GRH; *φ*1+1 underlined, collinear hydrophobic residues italicized) isolated from a phage-display screen using BBD as bait. However, because of its simple methyl side chain, it is likely that this alanine residue permits adoption of the required local structure and “space” for binding via other BBD/BH3-residue interactions rather than being involved directly in binding. Thus, while binding was abrogated by mutation of this alanine to most other collinear residues of non-binding BH3s, mutation to cysteine or serine (present in BH3s of Bok and Bax, respectively) was compatible with binding to BBD. Also, introduction of this alanine along with adjacent glutamine and glutamate residues (as present in Bim) into BBD-refractory Bax BH3 was sufficient to confer BBD interaction, although the binding was weaker than that of Bid BH3. Taken together, our data indicate that the *φ*1+1 alanine appears to be preferred and important for BH3 interaction, but that it is unlikely to contribute directly to binding and that other residues, in appropriate context, are directly involved in BH3 association and specificity. In regard to specificity of BH3 binding, we have established definitively that while BBD can recognize Bim and Bid BH3 domains, the former is not targeted by Bid BH3-B. Alignments of BBD and BH3-B, although revealing significant similarities, with three identical and three highly-related residues within the BH3-binding core regions, also show considerable divergence within and outside these central sequences (Fig. 1). It is noteworthy that previous di-alanine scanning mutagenesis of the 174–183 region of BBD identified residues within 174–181 as essential for interaction with Bim-BH3 [23]. Interestingly, however, combined mutation of the first two conserved hydrophobic residues, L_174_(qe)I_177_, did not abrogate binding (unpublished data), although identical or related hydrophobic residues are conserved in BH3 domains and are key contributors to the amphipathicity of the predicted α-helix. Single-residue mutagenesis across BBD, to alanine or to collinear BH3-B residues would be warranted to further delineate those amino acids and associated properties contributing to interaction with BH3 domains and to Bim- and Bid-BH3 selectivity.

In summary, data presented here identify similarity between vIRF-1 BBD and Bid BH3-B, corresponding inhibitory interactions of vIRF-1 BBD with the BH3-domains of Bim and Bid (both induced during and inhibitory to HHV-8 productive replication), and additional BBD binding of the BH3 domains of BOPs Bik, Bmf, Hrk and Noxa. To our knowledge, this is the first example of BOP targeting and inhibition via Bid BH3-B domain mimicry and thereby our data reveal a novel mechanism of viral evasion from host cell, apoptosis-mediated defense against viral infection and replication.

## Materials and Methods

### Cell culture, transfections and viral infection

Telomerase-immortalized endothelial (TIME) cells [46] and genetically engineered derivatives were cultured in EGM-2 MV medium (Lonza; Walkersville, MD) containing 5% fetal bovine serum (FBS) and cytokine supplements. TIME cell lines expressing vIRF-1 or RTA in doxycycline (Dox)-inducible fashion were generated using the Retro-X Tet-On Advanced system (Clontech Laboratories; Mountainview, CA). Briefly, the pRetroX-Tet-On Advanced plasmid was transfected into Phoenix cells and the supernatant was used to transduce TIME cells which were selected in G418 (400 µg/ml) to obtain TIME/Tet-On cells. The RTA coding region was derived from an existing eukaryotic expression vector as an *Eco*R1 restriction fragment and ligated into the *Eco*R1 site in the pRetroX-Tight-Pur plasmid. This was transfected into Phoenix cells, virus-containing supernatant used to infect TIME/Tet-On cultures, and transduced cells (TIME-TRE/RTA) selected in puromycin (1 µg/ml). Cloning discs were used to isolate individual colonies, and derived cells were screened by immunofluorescence assay for RTA expression following Dox induction. HeLa, HEK293, and HEK293T cells were maintained in Dulbecco's modified Eagle's medium supplemented with 10% FBS and gentamicin. BCBL-1/TRE-RTA [50] cells were maintained in RPMI 1640 medium containing 15% FBS and gentamicin. HHV-8 virus stocks were derived from doxycycline induced BCBL-1/TRE-RTA cultures and used to infect TIME cells as described previously [23]. For cell viability or immunofluorescence assays, cells were transfected using Fugene 6 (Roche Applied Science; Indianapolis, IN). For immunoprecipitation, cells were transfected using standard calcium-phosphate method or Lipofectamine 2000 (Invitrogen; Carlesbad, CA). For reporter assays, HEK293 cells were transiently transfected with plasmids expressing vIRF-1 and p53 along with the PG13-luc reporter vector (Addgene; Cambridge, MA) for 24 h and then lysed with passive lysis buffer (Promega; Madison, WI). Luciferase activity was measured by standard methods using D-luciferin and luminometry. For lentivirus production, HEK293T cells were transfected with virus vector and gag/pol-encoding plasmids using standard calcium-phosphate precipitation and virus was harvested after 48 h by centrifugation at 49,000×g. Cells were transduced with lentivirus in the presence of 5 µg/ml polybrene for 12 h and then cultured in complete media.

### Plasmids

For bacterial expression of T7-tagged proteins, coding sequences of T7 were first cloned between the *Nco*I and *Sal*I sites of pTYB4 (New England Biolabs; Ipswich, MA); coding sequences of vIRF-1 or enhanced green fluorescent protein (EGFP) were then inserted between the *Sal*I and *Sma*I sites of pTYB4-T7. The EGFP cDNA was amplified from vector pEGFP N1 (Clontech Laboratories). For bacterial expression of EGFP-fused BH3 peptides, EGFP coding sequences were inserted between the *Sal*I and *Eco*RI sites of pTYB4 and BH3 sequences (ds-oligonucleotides) were then inserted between the *Nhe*I and *Sal*I sites of pTYB4-EGFP. For the bacterial expression of *Discosoma* red fluorescent protein (DsRed)-fused peptides, coding sequences for DsRed were amplified from vector pDsRed2 (Clontech Laboratories) and inserted between the *Sal*I and *Eco*RI sites of pTYB4-T7. Coding sequences of vIRF-1 Bim-binding domain (BBD) or Bid BH3-B domain were inserted between the *Eco*RI and *Sma*I sites of pTYB4-T7-DsRed. The BBD sequence was also inserted between the *Bam*HI and *Eco*RI sites of pGEX4T-1 (GE Healthcare Life Sciences; Piscataway, NJ) for expression of GST-BBD. For generation of recombinant S-tagged tBid (see below), the coding sequence of tBid (comprising codons 61–195 of Bid_L_ [64]) was inserted between the *Bam*HI and *Eco*RI sites of pET-32a(+) (Novagen; Madison, WI). BOP and Bcl-2 cDNA sequences linked to Flag were cloned between the *Bam*HI and *Eco*RI site of pcDNA3.1 (Invitrogen) for expression in transfected cells. Plasmids expressing vIRF-1 and Bim were described previously [23]. Mutagenesis was performed by a PCR-mediated method using *Pfx* DNA polymerase (Invitrogen) with oligonucleotide primers containing deletion or substitution mutations. Two short hairpin RNAs (shRNA) for Bid were cloned into pYNC352/puro (a derivative of pTYB6 [65], [66]) using *Bam*HI and *Mlu*I enzyme sites. The target sequences of the shRNAs correspond to 5′- GGGATGAGTGCATCACAAACC -3′ (sh1) and 5′-CTTTCACACAACAGTGAATTT-3′ (sh2).

### Antibodies

Commercially obtained antibodies used in this study were as follow: T7, Novagen (Madison, WI), catalog number 65922; GFP, Bcl-2 and cytochrome c, Epitomics (Burlingame, CA), catalog numbers 1533-1, 1017-1 and 1896-1; Flag M2 and β-actin, Sigma (St. Louis, MI), catalog numbers F3165 and A5441; polyclonal Flag and Bim antibodies, Cell Signaling Technology (Beverly, MA), catalog numbers 2368 and 2819; Bax (N-20, catalog number sc-493), Bid (5C9, sc-56025), GST (B-14, sc-138), prohibitin (H-80, sc-28259), TOM20 (F-10, sc-17764), VDAC1 (20B12, sc-58649), lactate dehydrogenase (H-160, sc-33781), and histone deacetylase 1 (H-11, sc-8410) antibodies were purchased from Santa Cruz Biotechnologies (Santa Cruz, CA); LANA (LN53), Advanced Biotechnologies Inc. (Columbia, MD), catalog number 13-21-100. vIRF-1 rabbit antiserum was provided by Dr. Gary Hayward.

### Immunoblotting and immunofluorescence

For immunoblotting, cells were lysed in lysis buffer (50 mM Tris-HCl [pH 8.0], 150 mM NaCl, 1 mM EDTA, 1% IGEPAL CA-630, and 0.25% sodium deoxycholate) freshly supplemented with protease inhibitor cocktail (Sigma) for 1 h on ice. After centrifugation at 12,000×g for 20 min, the supernatant was used as a whole cell extract. For immunoblotting, proteins were size fractionated by sodium dodecyl sulfate-polyacrylamide gel electrophoresis (SDS-PAGE) and transferred to a nitrocellulose or polyvinylidene fluoride membranes. Immunoreactive bands were detected with enhanced chemiluminescence solution (GE Healthcare Life Sciences) and visualized on X-ray film or digitally using a chemiluminescence imager. For immunofluorescence assays, cells were grown on a 0.1% gelatin-coated coverglass or a chamber slide and were fixed and permeabilized in chilled methanol. Following incubation with Superblock blocking buffer in phosphate-buffered saline (PBS) (Thermo Scientific Inc.; Rockford, IL), coverslips were incubated with primary antibody, washed with PBS, and then incubated with appropriate fluorescent dye-conjugated secondary antibody. The coverslips were mounted in 90% glycerol in PBS containing 10 mg/ml p-phenylenediamine, an antifade reagent.

### Co-precipitation assays

For immunoprecipitation, HEK293T cells transfected with plasmids encoding vIRF-1 or Flag epitope-tagged BH3-only proteins (BOPs) were lysed in lysis buffer, and cell extracts were incubated with anti-Flag M2 affinity gel (Sigma) for 3 h at 4°C. After washing with lysis buffer, immune-complexes were eluted with 30 µl of 3× Flag peptide (150 ng/µl), subjected to SDS-PAGE, and analyzed by immunoblotting using vIRF-1 antiserum or polyclonal Flag antibody. For *in vitro* binding assays, fluorescent protein-fused peptides or T7-tagged proteins expressed in *E. coli* from vector pTYB4 (New England Biolabs) were purified according to the manufacturer's protocol. Bacterially expressed glutathione-S-transferase (GST)-fused proteins were purified by standard methods. 0.5 µg of EGFP or EGFP-BH3, fused to intein-chitin binding domain (CBD) and immobilized on chitin beads, was mixed with 1 µg of purified T7-DsRed-fused BBD or BH3-B peptides and incubated for 1 h at room temperature. After washing four times with Tris-buffered saline (TBS) supplemented with 0.1% Tween 20, bead-associated proteins were size-fractionated by SDS-PAGE and analyzed by immunoblotting using T7- or GFP-specific antibodies. To screen for BBD interaction with a variety of BOP BH3 domains, 1 µg of GST or GST-BBD protein immobilized on glutathione sepharose 4B beads was mixed with 2 µg of EGFP-BH3 fusion proteins. GST-BBD and its associated proteins were eluted with 10 mM reduced glutathione in TBS, size-fractionated by SDS-PAGE, and analyzed by immunoblotting using GFP- or GST-specific antibodies.

### Subcellular fractionation and associated assays

For nucleo-cytoplasmic fractionation, cells were homogenized in buffer A (20 mM Tricine-KOH [pH 7.8], 5 mM MgCl_2_, 25 mM KCl, 0.25 M sucrose, and protease inhibitor cocktail) using a Dounce homogenizer. After centrifugation at 1,000×g for 10 min, the supernatant was used as the cytoplasmic fraction, and the pellet was subjected to 25–35% iodixanol discontinuous gradient centrifugation. Nuclei were collected from the interface between 30 and 35% iodixanol and resuspended in buffer C (20 mM HEPES [pH 8.0], 1.5 mM MgCl_2_, 420 mM NaCl, 0.2 mM EDTA, and protease inhibitor cocktail). For *in vitro* cytochrome c release assays, mitochondria were isolated by sucrose density gradient centrifugation. Briefly, HEK293T cells grown to subconfluence in a 10 cm dish were washed in ice-cold PBS and resuspended in mitochondrial isolation buffer (MIB: 210 mM mannitol, 70 mM sucrose, 1 mM EDTA, and 10 mM HEPES [pH 7.5]) supplemented with protease inhibitor cocktail. Next, cells were homogenized with 40 strokes of Dounce homogenizer and centrifuged at 2,000×g for 10 min. The supernatant was further centrifuged at 13,000×g at 4°C for 10 min. After resuspending in MIB, the resulting pellet was layered on top of a discontinuous sucrose gradient consisting of 1.2 M sucrose in buffer H (10 mM HEPES [pH 7.5], 1 mM EDTA, and 0.1% BSA) on top of 1.6 M sucrose in buffer H. Following centrifugation at 131,000×g for 2 h at 4°C, mitochondria were recovered at the 1.6–1.2 M sucrose interface, washed in MIB, centrifuged at 13,000×g at 4°C for 10 min, and resuspended in 100 µl of MIB. For other studies, mitochondria were purified using Axis-Shield OptiPrep (Sigma) according to the manufacturer's protocol. For proteinase K treatment, mitochondria in MIB (0.5 mg/ml) were incubated on ice for 30 min with 20 µg/ml proteinase K; the reaction was stopped by the addition of 2 mM PMSF, and mitochondria were then washed twice in 1 ml of MIB and resuspended in MIB or MRM buffer (250 mM sucrose, 10 mM HEPES [pH 7.5], 1 mM ATP, 5 mM sodium succinate, 80 µM ADP, 2 mM K_2_HPO_4_). For mitochondrial binding assays, 1 µg of recombinant T7-EGFP or T7-vIRF-1 protein was added to the proteinase K-pretreated or untreated mitochondria in MRM buffer (50 µg protein/50 µl) supplemented with PMSF and 0.1 mg/ml BSA. The mixtures were incubated for 1 h at 30°C and then centrifuged at 12,000×g for 5 min. The mitochondrial pellets were washed twice in MRM buffer, and the final washed pellets were resuspended in SDS sample buffer. Trichloroacetic acid (TCA, 10% final concentration) was added to the supernatants to precipitate proteins prior to SDS-PAGE and immunoblot analysis. For *in vivo* cytochrome c release assay, transfected HEK293T cells were subjected to Dounce homogenization (30 strokes) in MIB buffer. The homogenate, an aliquot of which was used as a total cell extract, was centrifuged at 1000×g for 10 min at 4°C to remove nuclei and unbroken cells. The resulting supernatant was centrifuged at 100,000×g for 1 h at 4°C to yield the final soluble cytosolic fraction (S100).

### *In vitro* cytochrome c release assay

*In vitro* mitochondrial permeabilization assays based on cytochrome c release were undertaken essentially as described by Arnoult [67] and outlined below. Thioredoxin/His_6_/S-tagged tBid protein encoded from pET-32a(+) was purified using Ni-NTA His-tag affinity chromatography, and S-tBid (100 µg/ml) was eluted following thrombin (0.5 unit/ml) treatment for 2 h at room temperature. Thioredoxin and His sequences were retained on the Ni-NTA resin. S-tBid was purified away from thrombin using protein S agarose, eluted with 3 M MgCl_2_, and dialyzed against dilution buffer (25 mM HEPES-KOH [pH 7.4], 0.1 M KCl). Following preincubation of 10 nM or 100 nM of S-tBid with or without 500 nM of GST-fusion peptides or 100 nM of T7-vIRF-1 proteins (wild-type or mutated) in dilution buffer supplemented with 1 mg/ml of fatty acid-free bovine serum albumin (FA-BSA), purified mitochondria were added (50 µg protein/50 µl of mitochondrial buffer: 125 mM KCl, 0.5 mM MgCl_2_, 3 mM succinic acid, 3 mM glutamic acid, 10 mM HEPES-KOH [pH 7.4], 1 mg/ml FA-BSA, and protease inhibitor cocktail). The reaction mixtures were incubated at 30°C for 30 min and then centrifuged at 12,000×g for 5 min at 4°C to pellet the mitochondria. The supernatants were quickly removed, and the pellet was resuspended in 70 µl of mitochondria lysis buffer (50 mM Tris-HCl [pH 7.4], 150 mM NaCl, 2 mM EDTA, 2 mM EGTA, 0.2% Triton X-100, and 0.3% IGEPAL CA-630). The amounts of cytochrome c in the supernatant and pellet fractions were determined by immunoblotting.

### Annexin V apoptosis assay

After plasmid transfection of 293T cells on a coverslip, the cells were stained with Cy3-conjugated annexin V (Biovision Inc.; Mountain View, CA) in AV binding buffer (10 mM HEPES [pH 7.4], 140 mM NaCl, and 2.5 mM CaCl_2_), fixed with 2% formaldehyde in AV binding buffer for 10 min, and mounted in glycerol medium containing DAPI. Annexin V-positive cells, fluorescent under UV, were counted from three randomly selected low-magnification microscopic fields.

### Screening of phage display library

The Ph.D.-12 library, comprising a complexity in excess of one billion independent clones, was purchased from New England Biolabs (Beverly, MA). Screening was performed by a solution-phase panning method with affinity bead capture as described by the manufacturer's protocol. In brief, a mixture of purified GST-BBD (500 nM) and the library (2×10^11^ pfu) was incubated for 20 min at room temperature and added to glutathione 4B beads pre-blocked with BSA. After incubating for 15 min and washing the mixture with Tris-buffered saline (TBS) containing 0.1% Tween 20, phage were eluted and amplified. Negative selection using purified GST was performed prior to the second round of panning. After the third round of panning, the mixture was washed with TBS containing 0.5% Tween 20, and phage were eluted and plaque-purified prior to DNA preparation and sequencing.

### HHV-8 replication

For HHV-8 infection, TIME cells were centrifuged at 1,000×g for 1 h in the presence of HHV-8 virions and then cultured in fresh complete medium for 7 days to allow establishment of latency in the absence of ongoing lytic replication. After lentiviral transduction of control (NS), Bid, or Bim shRNAs for 2 days into HHV-8^+^ TIME cells, lytic replication of HHV-8 was induced by treatment with TPA (20 ng/ml) or 1 µg/ml doxycycline (TIME-TRE/RTA cells). For determination of encapsidated HHV-8 genome copy number, viral DNA was isolated using standard phenol extraction and glycogen/ethanol precipitation methods following pre-treatment of virus suspensions with DNaseI for 20 min at 37°C to remove any unencapsidated DNA. For the determination of the viral genome copy number, all qPCRs were performed in a 96-well microplate using an ABI Prism 7500 detection system (Applied Biosystems; Foster City, CA) with SYBR green/ROX master mix (SuperArray Bioscience Corp.; Frederick, MD). For induced TIME-TRE/RTA cells, infectious virus titers were measured by application of induced culture media-derived virus to naïve TIME cells and immunofluorescence staining for HHV-8 latency-associated nuclear antigen (LANA).

## Supporting Information

Figure S1 **Characterization of TIME-TRE/RTA cells line.** (A) TIME cells transduced with tetracycline-responsive repressor/transactivator (rtTA) expression cassette and rtTA-responsive RTA expression cassette (see Materials and Methods) were isolated as clonal cell lines and tested by immunofluorescence assay for RTA expression following treatment with doxycycline (Dox, 1 µg/ml) for 24 h. An example of analysis of one cell line, which was used in subsequent studies, is shown. (B) RTA expression in response to different concentrations of Dox (applied for 15 h) was analyzed by immunoblotting of SDS-PAGE fractionated cell extracts using RTA-specific antiserum. Antibody to β-actin was used for immunoblotting to confirm equivalent protein loading. (C) TIME-TRE/RTA cells were infected with HHV-8 r219 (Vieira & O'Hearn; Virology 325:225–240), which expresses GFP constitutively and RFP under the control of a lytic cycle promoter, the latter providing a marker of lytic induction. The cells were allowed to rest for 5 days to ensure establishment of latency and absence of residual lytic replication. These cells expressed GFP in ∼100% of cells, and very few (<1%) expressed RFP. Parallel cultures of these latently infected TIME-TRE/RTA cells were either left untreated or were treated with Dox (1 µg/ml) for the indicated times and then visualized under UV microscopy for detection of RFP^+^ cells. (D) An analogous experiment was undertaken using BCBL-1 culture-derived HHV-8, but here immunofluorescence staining for K8.1-encoded late lytic antigen was used to detect cells supporting productive replication. In this experiment, application of Dox was either sustained for 1 or 5 days prior to fixation and immunofluorescence staining or applied for 2 days and then removed prior to IFA analysis 5 days post-induction. For comparison, a parallel culture was treated with TPA (20 ng/ml) for five days prior to K8.1 immunostaining.
(TIF)

Figure S2 **Confocal immunofluorescence analysis of vIRF-1 and Bid colocalization to mitochondria.** HHV-8^+^ TIME-TRE/RTA cells were induced with doxycycline (1 µg/ml) for 48 hours, treated with mitochondrial-specific fluorescent marker [MitoTracker (Cy3, red); Invitrogen], and then fixed and immuno-stained (essentially as outlined in Materials and Methods) for vIRF-1 (Cy5, purple) and Bid (FITC, green) and counterstained with DAPI (nuclear, blue). Staining patterns for vIRF-1 and Bid colocalization varied from large structures (most likely representing fused or aggregated mitochondria) to very fine punctate staining corresponding with MitoTracker dye. Examples of triple vIRF-1, Bid and mitochondrial fluorescence are indicated by white arrows, spots of Bid and mitochondrial signals by yellow arrows, and vIRF-1 and mitochondria staining by mauve arrows. The 40× fields are derived from a single section; the 100× fields represent two successive sections (z1, z2).
(TIFF)
