# Supplementary figures and images for: Human Herpesvirus 8 Interferon Regulatory Factor-Mediated BH3-Only Protein Inhibition via Bid BH3-B Mimicry
Source: PLoS Pathog. 2012 Jun 7;8(6):e1002748. doi: 10.1371/journal.ppat.1002748 (PMC3369933; doi:10.1371/journal.ppat.1002748)

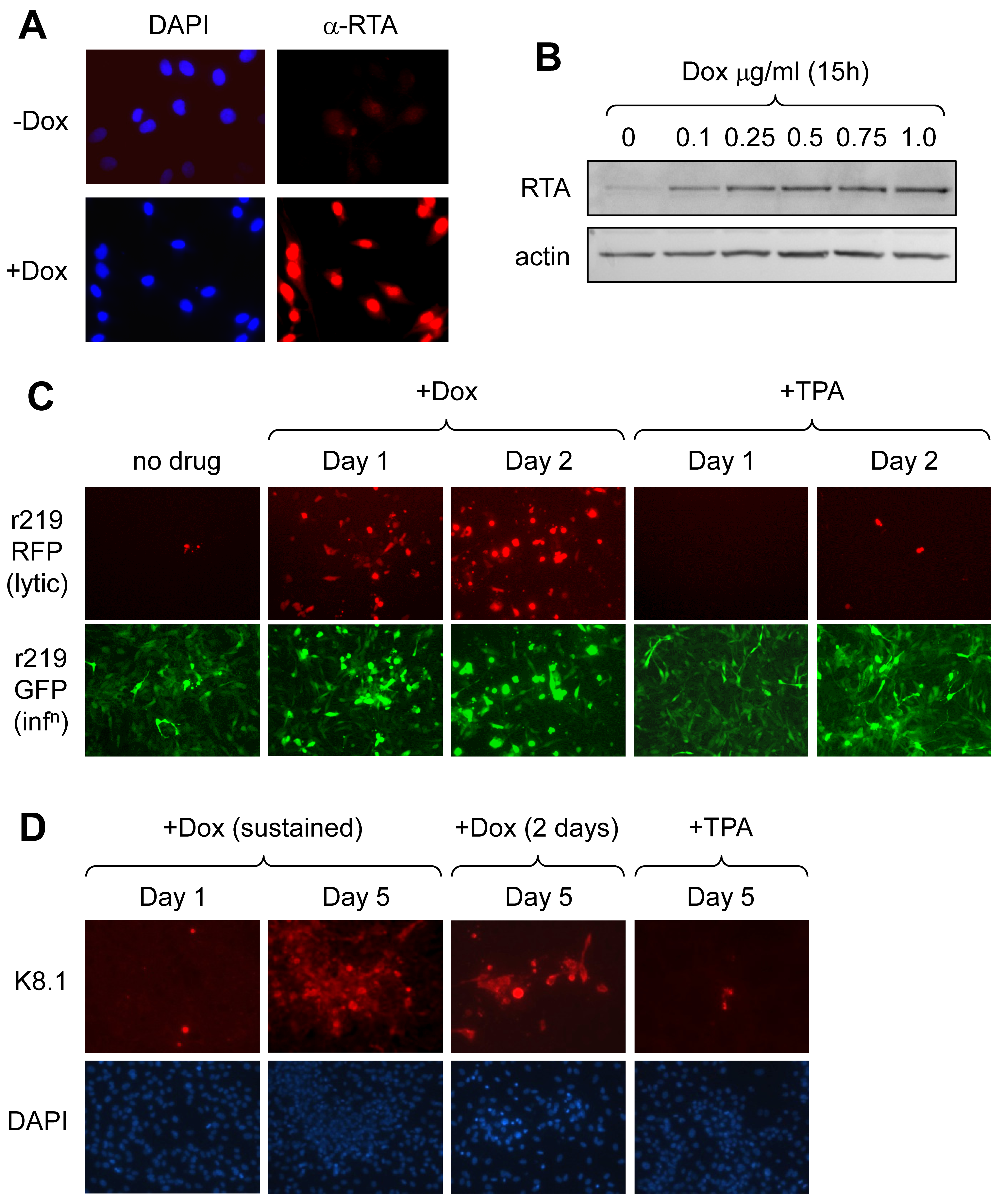

Supplement: Figure S1 — Characterization of TIME-TRE/RTA cells line. (A) TIME cells transduced with tetracycline-responsive repressor/transactivator (rtTA) expression cassette and rtTA-responsive RTA expression cassette (see Materials and Methods) were isolated as clonal cell lines and tested by immunofluorescence assay for RTA expression following treatment with doxycycline (Dox, 1 µg/ml) for 24 h. An example of analysis of one cell line, which was used in subsequent studies, is shown. (B) RTA expression in response to different concentrations of Dox (applied for 15 h) was analyzed by immunoblotting of SDS-PAGE fractionated cell extracts using RTA-specific antiserum. Antibody to β-actin was used for immunoblotting to confirm equivalent protein loading. (C) TIME-TRE/RTA cells were infected with HHV-8 r219 (Vieira & O'Hearn; Virology 325:225–240), which expresses GFP constitutively and RFP under the control of a lytic cycle promoter, the latter providing a marker of lytic induction. The cells were allowed to rest for 5 days to ensure establishment of latency and absence of residual lytic replication. These cells expressed GFP in ∼100% of cells, and very few (<1%) expressed RFP. Parallel cultures of these latently infected TIME-TRE/RTA cells were either left untreated or were treated with Dox (1 µg/ml) for the indicated times and then visualized under UV microscopy for detection of RFP+ cells. (D) An analogous experiment was undertaken using BCBL-1 culture-derived HHV-8, but here immunofluorescence staining for K8.1-encoded late lytic antigen was used to detect cells supporting productive replication. In this experiment, application of Dox was either sustained for 1 or 5 days prior to fixation and immunofluorescence staining or applied for 2 days and then removed prior to IFA analysis 5 days post-induction. For comparison, a parallel culture was treated with TPA (20 ng/ml) for five days prior to K8.1 immunostaining. (TIF) [file ppat.1002748.s001.tif]

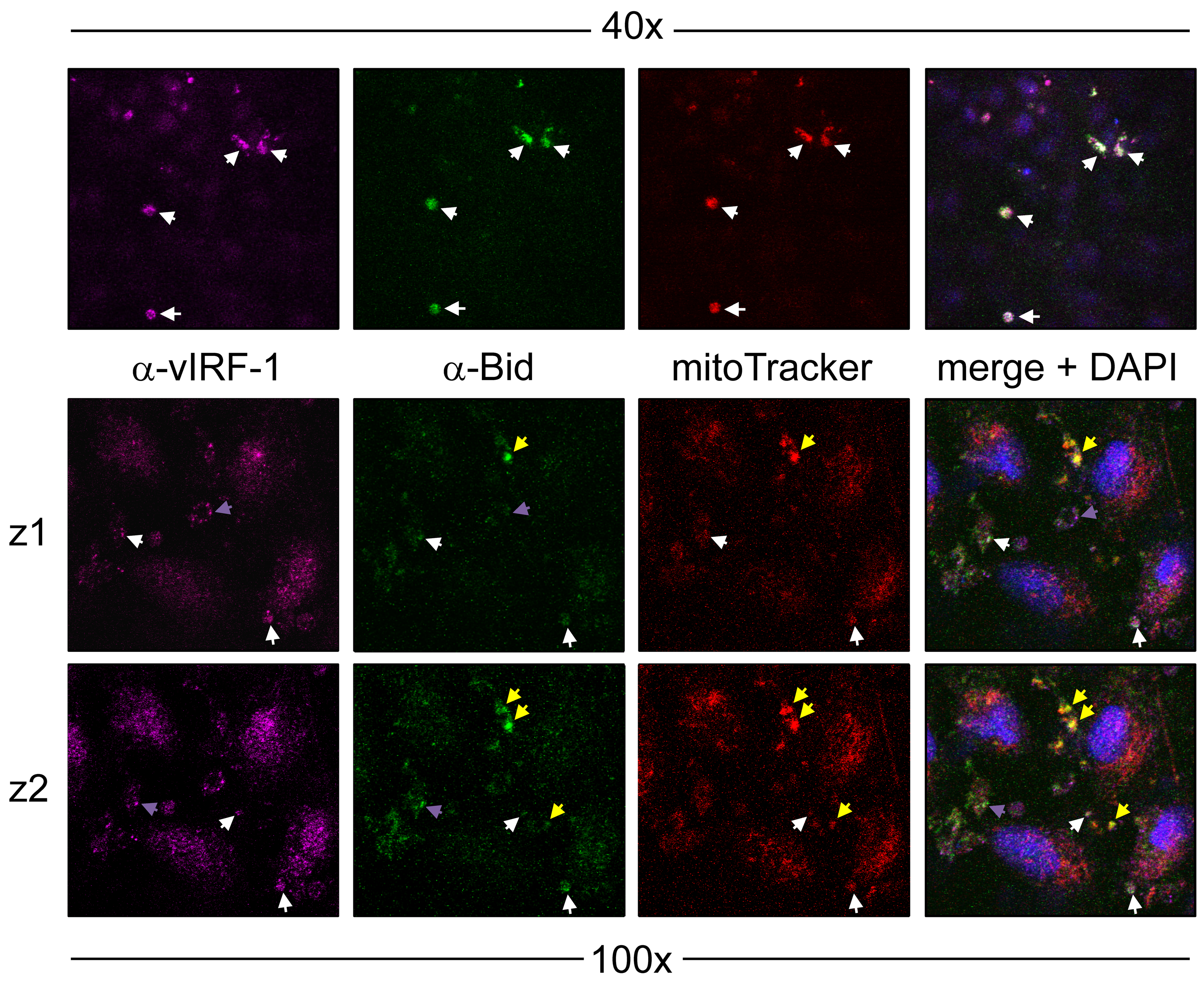

Supplement: Figure S2 — Confocal immunofluorescence analysis of vIRF-1 and Bid colocalization to mitochondria. HHV-8+ TIME-TRE/RTA cells were induced with doxycycline (1 µg/ml) for 48 hours, treated with mitochondrial-specific fluorescent marker [MitoTracker (Cy3, red); Invitrogen], and then fixed and immuno-stained (essentially as outlined in Materials and Methods) for vIRF-1 (Cy5, purple) and Bid (FITC, green) and counterstained with DAPI (nuclear, blue). Staining patterns for vIRF-1 and Bid colocalization varied from large structures (most likely representing fused or aggregated mitochondria) to very fine punctate staining corresponding with MitoTracker dye. Examples of triple vIRF-1, Bid and mitochondrial fluorescence are indicated by white arrows, spots of Bid and mitochondrial signals by yellow arrows, and vIRF-1 and mitochondria staining by mauve arrows. The 40× fields are derived from a single section; the 100× fields represent two successive sections (z1, z2). (TIFF) [file ppat.1002748.s002.tif]
